# Supplementary material for: Design, Synthesis and Biological Evaluation of Novel Coumarin-Based Hydroxamate Derivatives as Histone Deacetylase (Hdac) Inhibitors with Antitumor Activities
Source: Molecules. 2019 Jul 15;24(14):2569. doi: 10.3390/molecules24142569 (PMC6680717; doi:10.3390/molecules24142569)

Supplementary Information for

Design, Synthesis and Biological Evaluation of Novel Coumarin-Based Hydroxamate Derivatives as Histone Deacetylase (Hdac) Inhibitors with Antitumor Activities

Feifei Yang [1], Na Zhao [1], Jiali Song [1], Kongkai Zhu [1], Cheng-shi Jiang [1], Peipei Shan [2,*] and Hua Zhang [1,*]

[1] School of Biological Science and Technology, University of Jinan, Jinan 250022, China
[2] Institute for Translation Medicine, Qingdao University, Qingdao 266071, China
* Correspondence: bio_zhangh@ujn.edu.cn (H.Z.); shanpeipei@qdu.edu.cn (P.S.); Tel.: 053189736199 (H.Z.); 053282991791 (P.S.)

Received: 27 May 2019; Accepted: 13 July 2019; Published: 15 July 2019

Table of contents

NMR and HR-ESIMS spectra and HPLC analysis of compound **10a**……………………………S2–S3

NMR and HR-ESIMS spectra and HPLC analysis of compound **10b**……………………………S4–S5

NMR and HR-ESIMS spectra and HPLC analysis of compound **10c**……………………………S6–S7

NMR and HR-ESIMS spectra and HPLC analysis of compound **10d**……………………………S8–S9

NMR and HR-ESIMS spectra and HPLC analysis of compound **10e**…………………………… S10–S11

NMR and HR-ESIMS spectra and HPLC analysis of compound **11a**……………………………S12–S13

NMR and HR-ESIMS spectra and HPLC analysis of compound **11b**…………………………… S14–S15

NMR and HR-ESIMS spectra and HPLC analysis of compound **11c**……………………………. S16–S17

NMR and HR-ESIMS spectra and HPLC analysis of compound **11d**…………………………… S18–S19

NMR and HR-ESIMS spectra and HPLC analysis of compound **11e**…………………………… S20–S21

NMR and HR-ESIMS spectra and HPLC analysis of compound **12a**…………………………… S22–S23

NMR and HR-ESIMS spectra and HPLC analysis of compound **12b**………………………….... S24–S25

HDAC inhibition activity of compounds **10b-e, 11a-e** and **12a-b**……………………………….S26

Inhibition activity of compounds **10e** and **11d** on different HDAC isoforms ….……………S27-28

Anti-proliferative activities of compounds **10e** and **11d** against different cancer cell lines…S29

Compound 10a

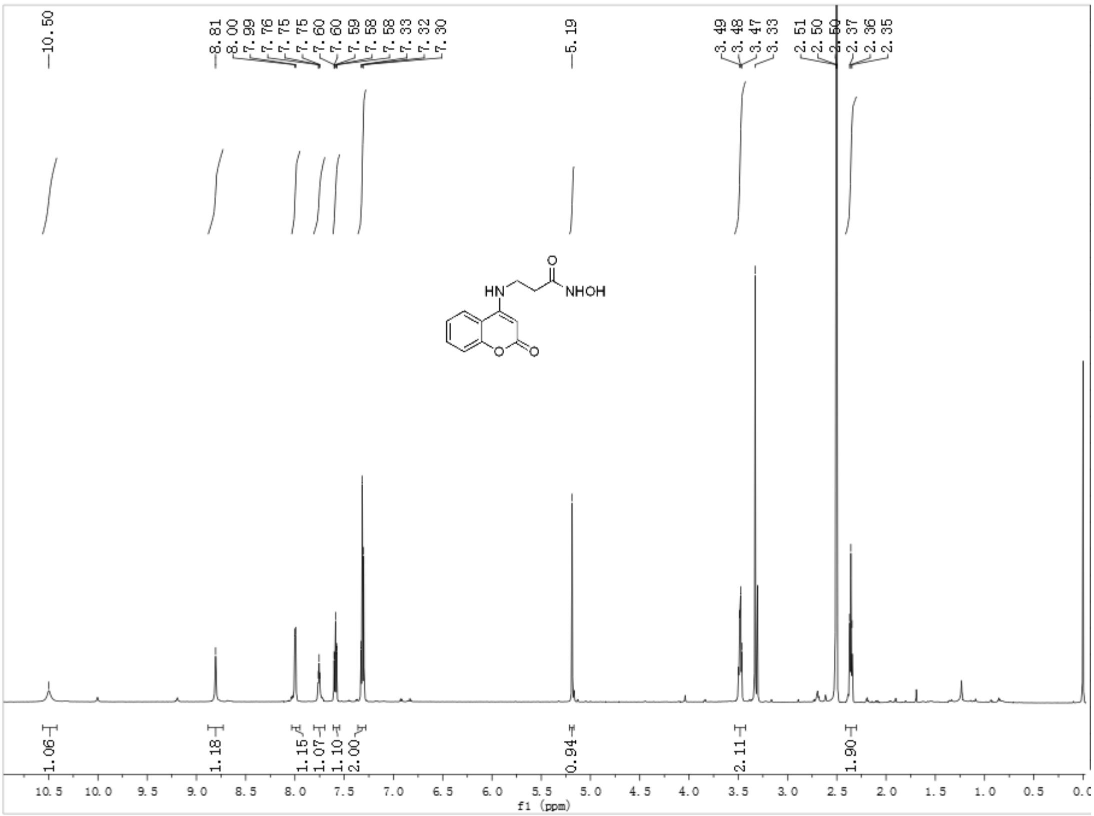

Fig. S1. The ^{1}H NMR spectrum for **10a**

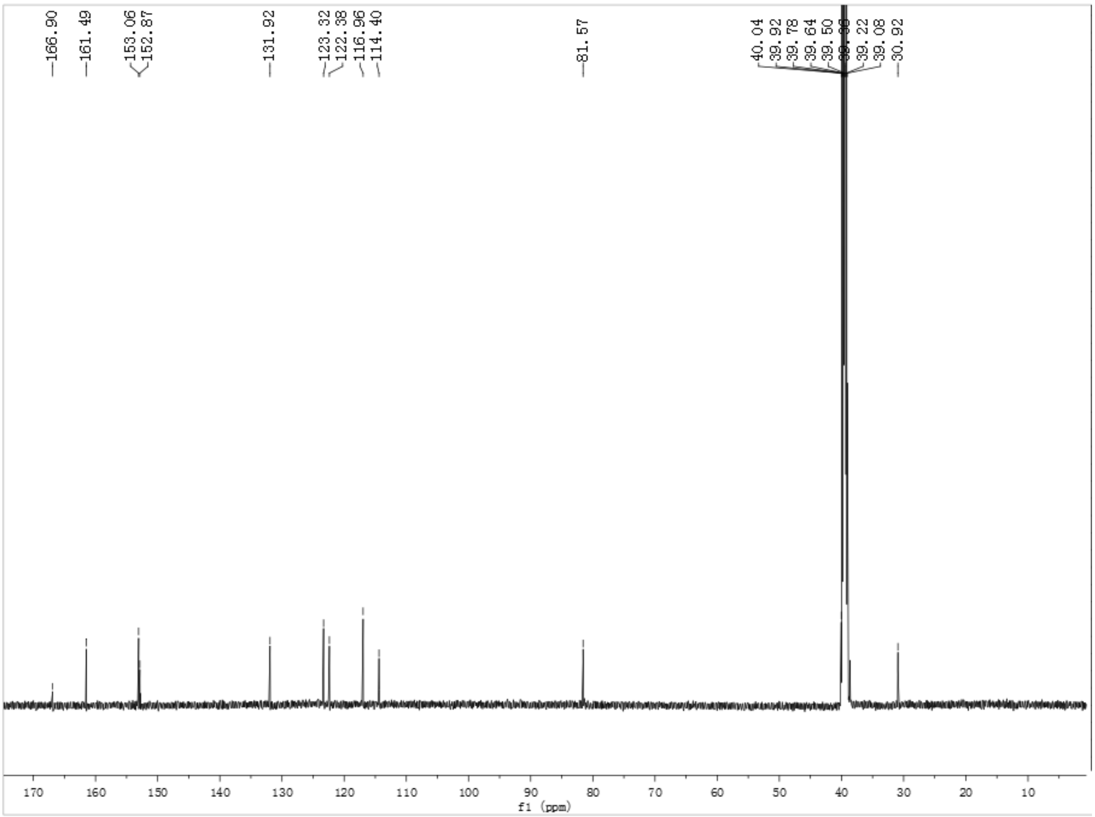

Fig. S2. The ^{13}C NMR spectrum for **10a**

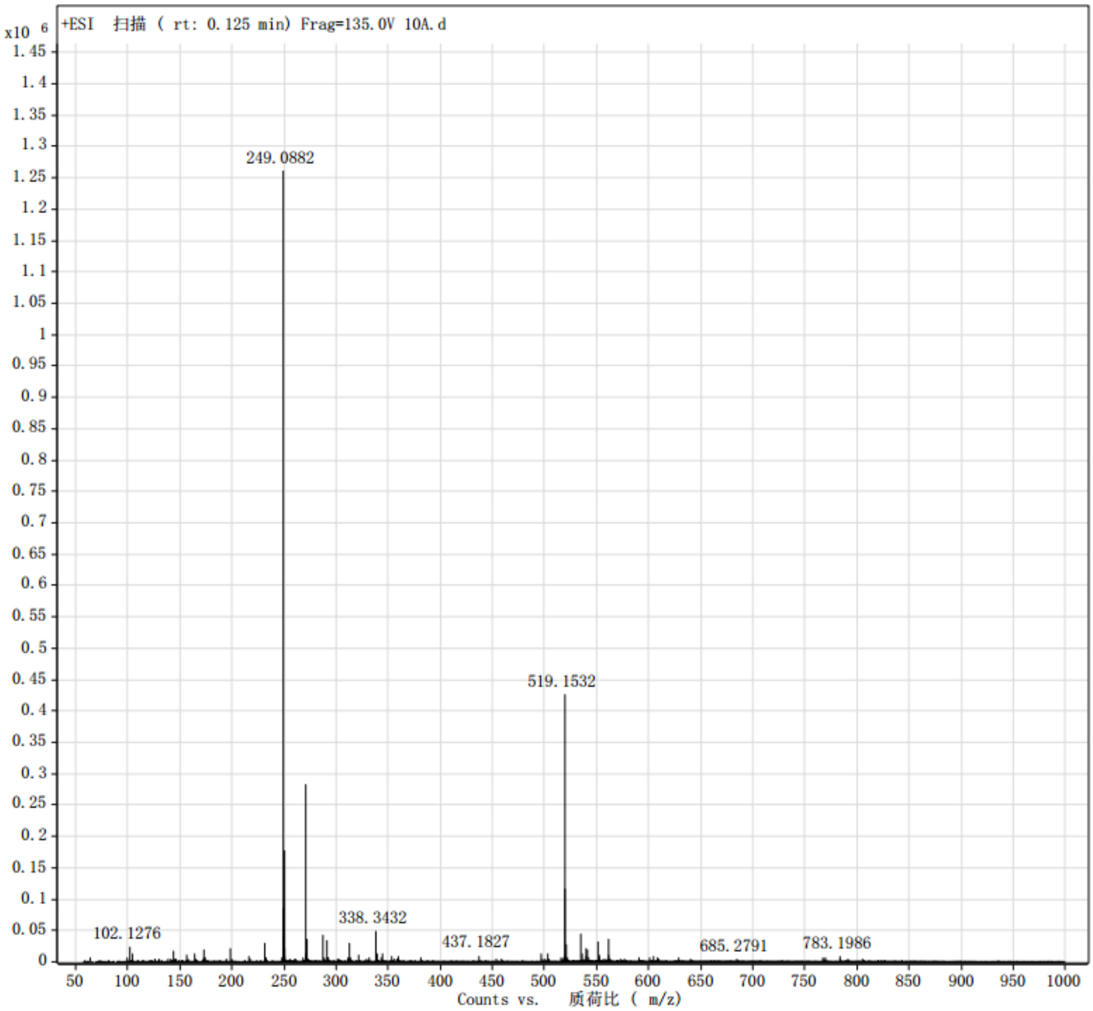

Fig. S3. The HR-ESIMS spectrum for **10a**

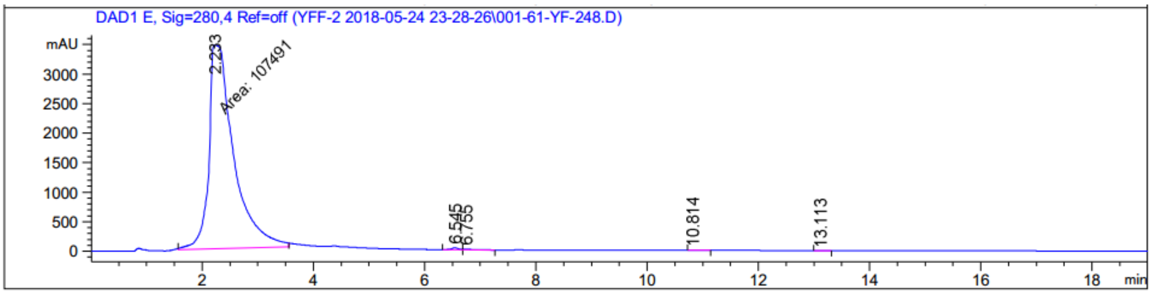

Peak #	RetTime [min]	Type	Width [min]	Area [mAU*s]	Height [mAU]	Area %
1	2.233	MM	0.5197	1.07491e5	3447.03101	99.6255
2	6.545	BV	0.1178	260.85858	33.14917	0.2418
3	6.755	VB	0.1452	115.08218	10.89933	0.1067
4	10.814	VB	0.1625	16.90411	1.40358	0.0157
5	13.113	BB	0.1169	11.17630	1.46718	0.0104

Fig. S4. The HPLC analysis for **10a**

Compound 10b

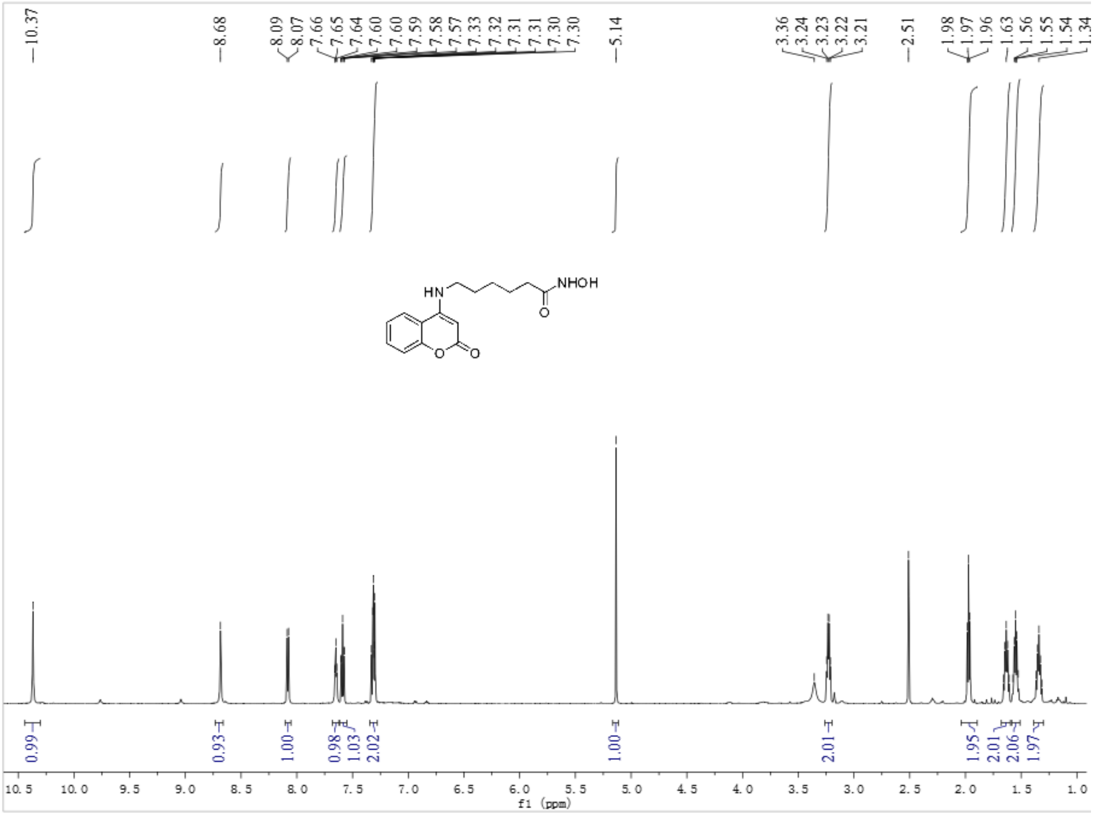

Fig. S5. The ^{1}H NMR spectrum for **10b**

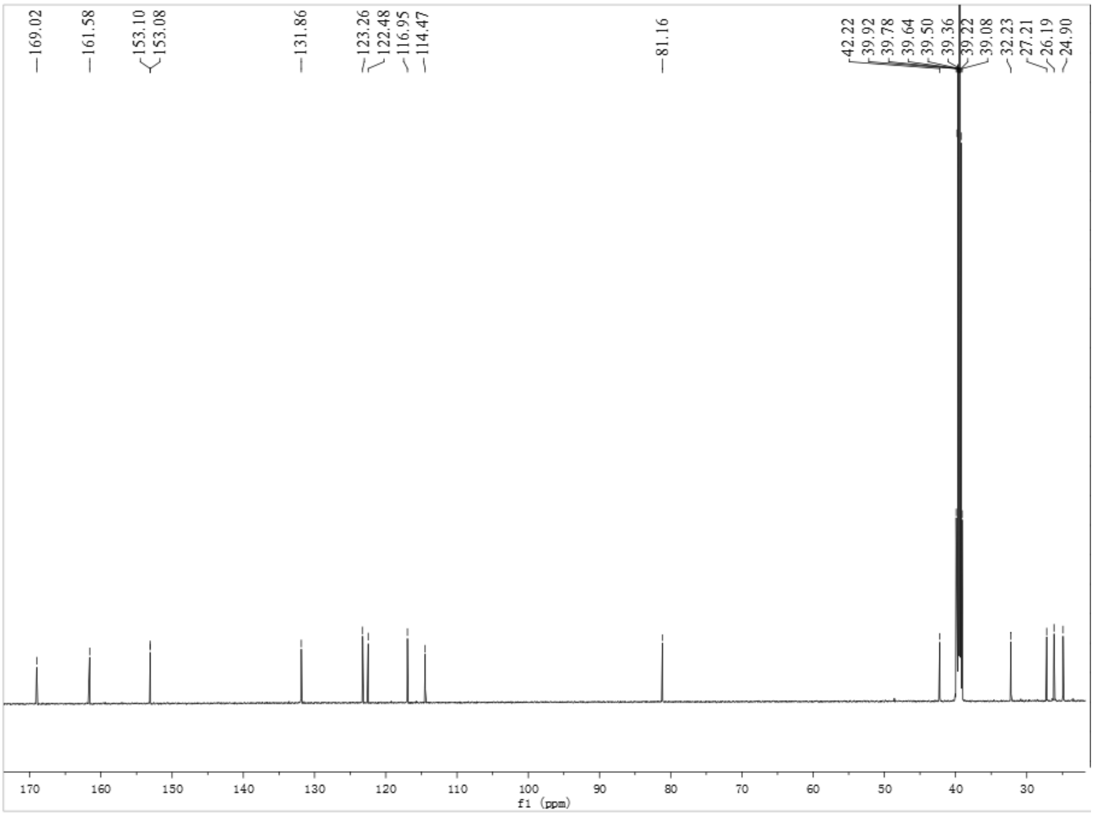

Fig. S6. The ^{13}C NMR spectrum for **10b**

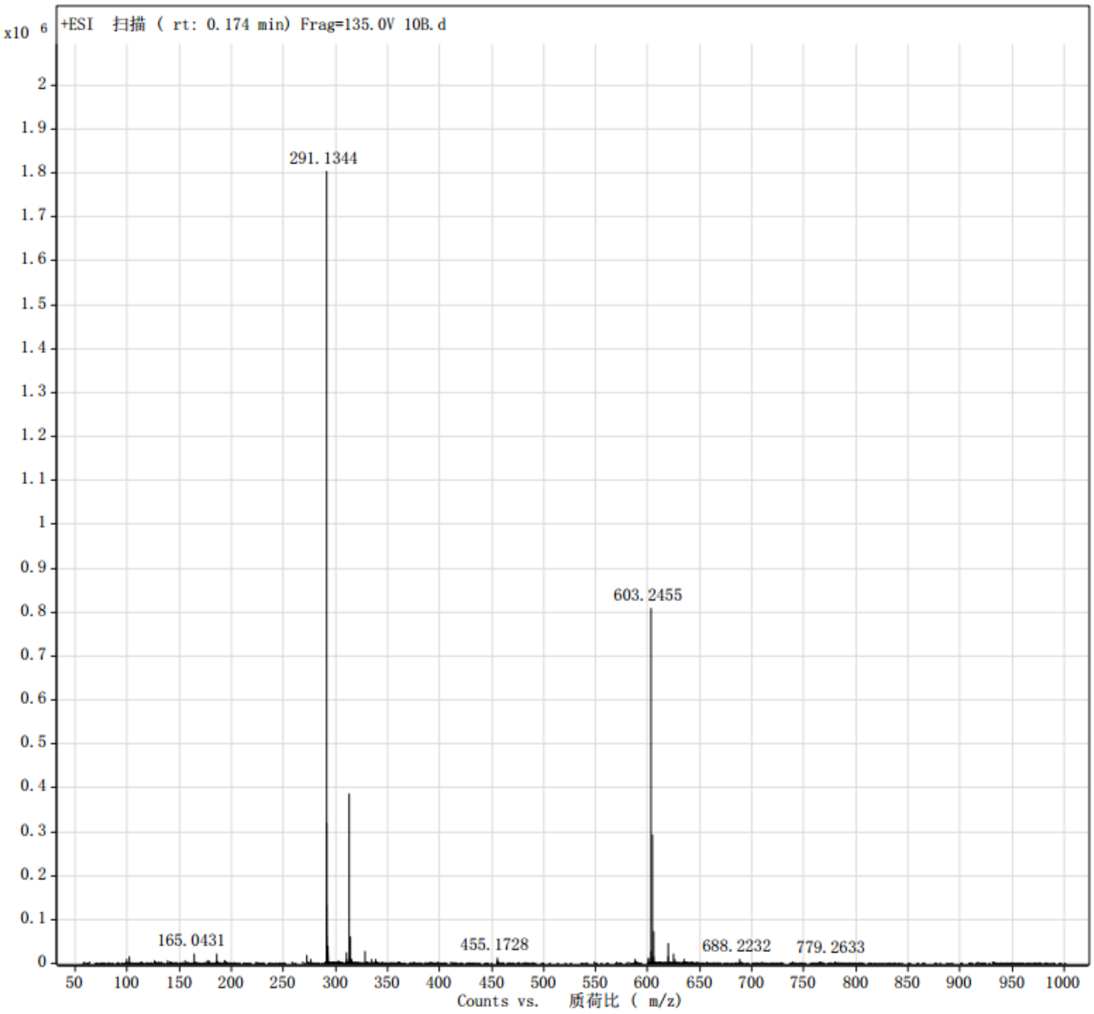

Fig. S7. The HR-ESIMS spectrum for **10b**

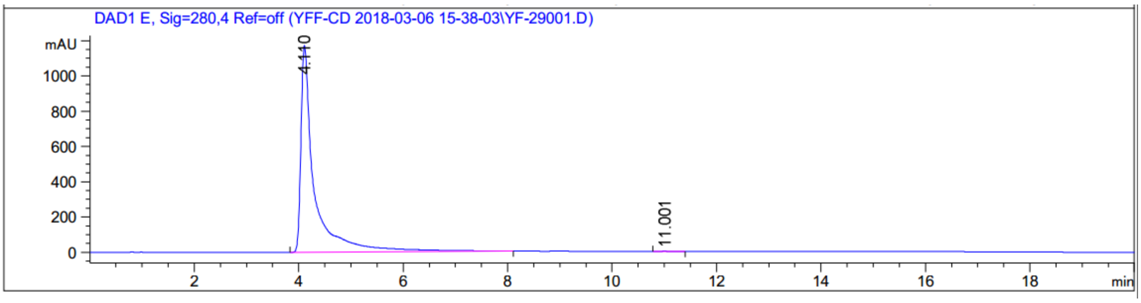

Peak #	RetTime [min]	Type	Width [min]	Area [mAU*s]	Height [mAU]	Area %
1	4.110	BB	0.2462	2.09069e4	1171.33508	99.8863
2	11.001	BB	0.1630	23.79875	2.18660	0.1137

Fig. S8. The HPLC analysis for **10b**

Compound 10c

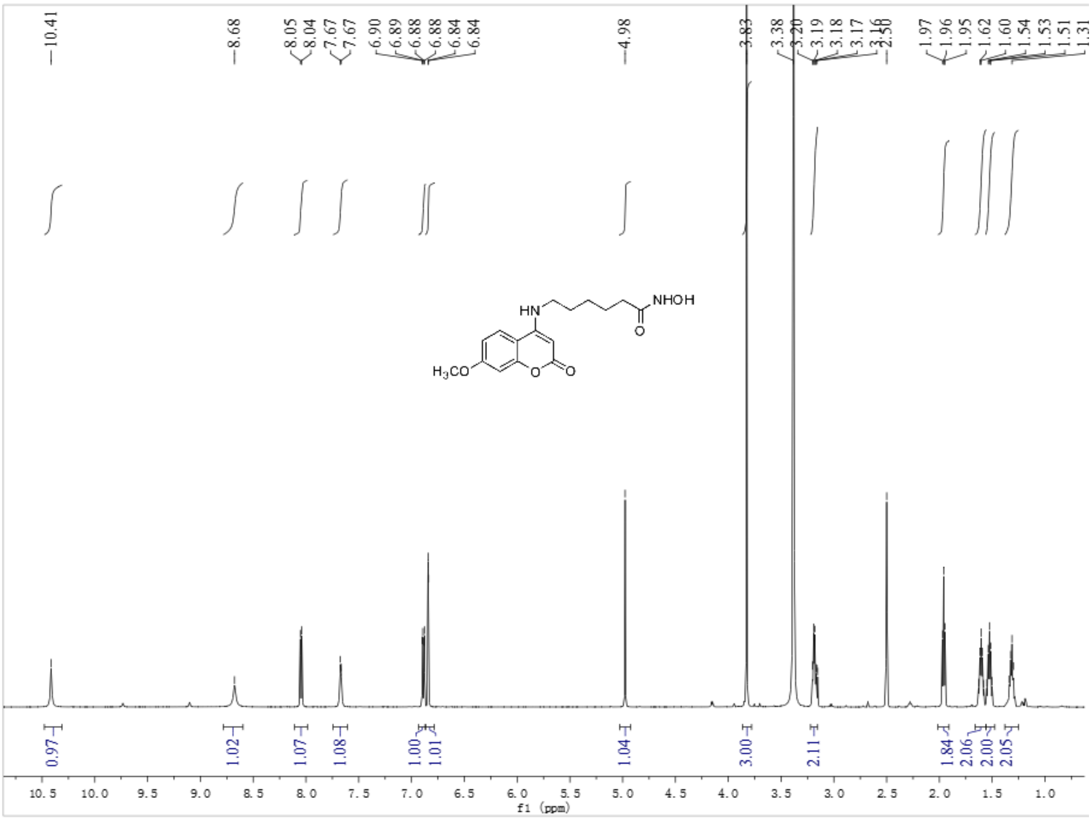

Fig. S9. The ^{1}H NMR spectrum for **10c**

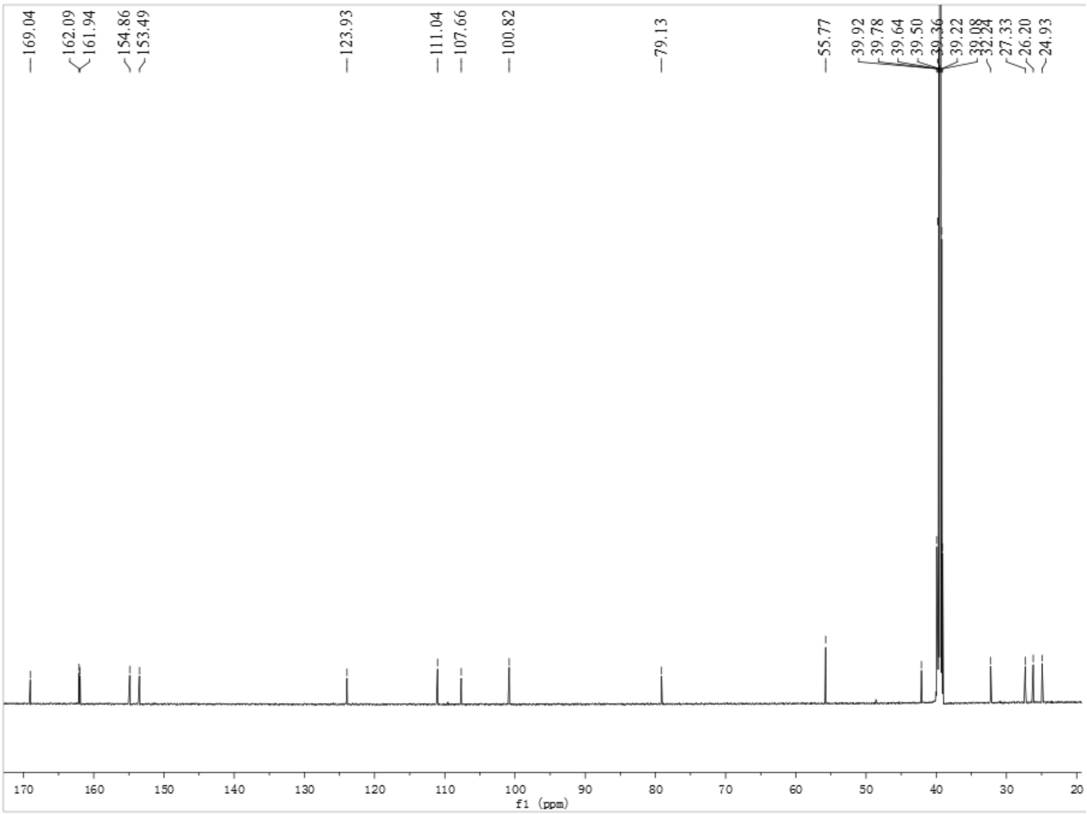

Fig. S10. The ^{13}C NMR spectrum for **10c**

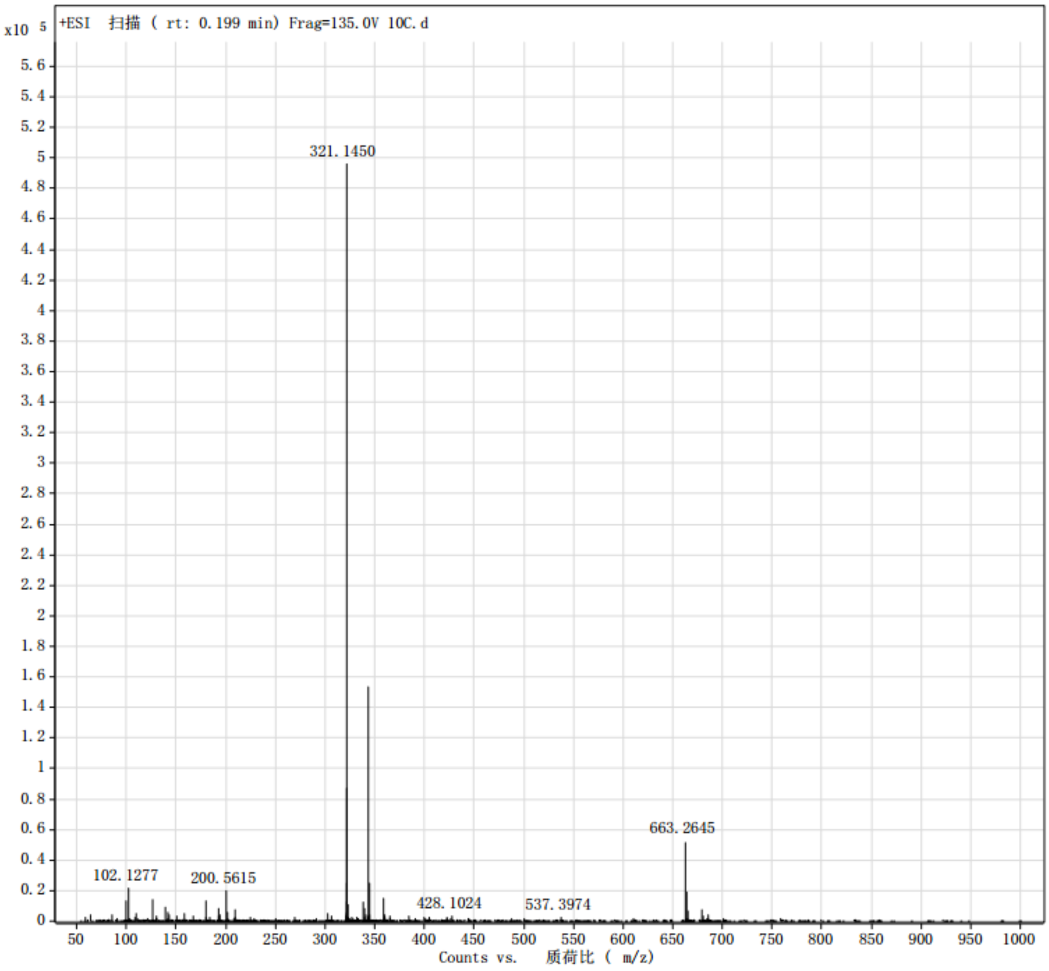

Fig. S11. The HR-ESIMS spectrum for **10c**

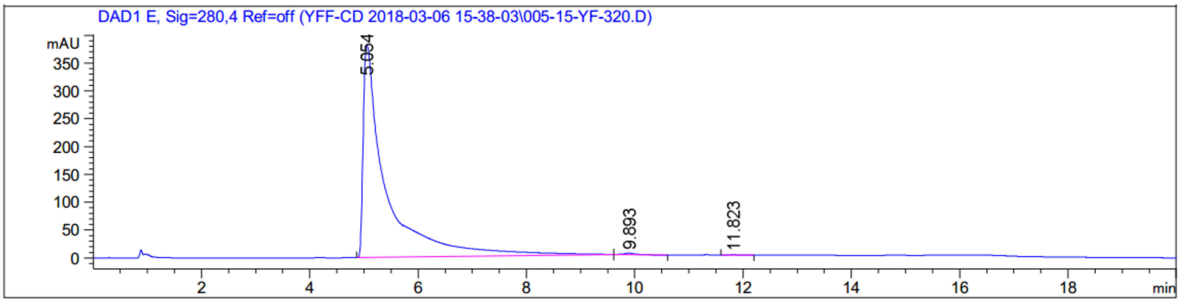

Peak #	RetTime [min]	Type	Width [min]	Area [mAU*s]	Height [mAU]	Area %
1	5.054	BB	0.3933	1.12422e4	382.08560	99.5442
2	9.893	BB	0.2125	37.81986	2.52915	0.3349
3	11.823	BB	0.1854	13.65597	1.08052	0.1209

Fig. S12. The HPLC analysis for **10c**

Compound 10d

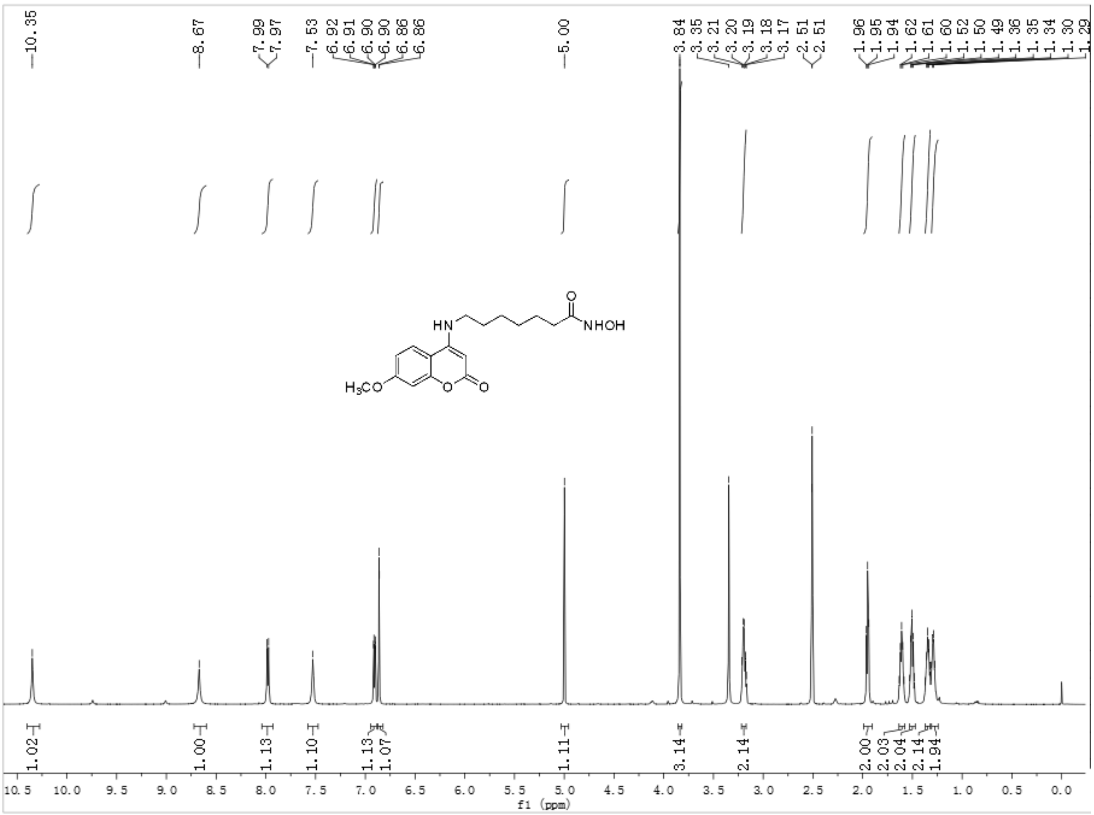

Fig. S13. The ^{1}H NMR spectrum for **10d**

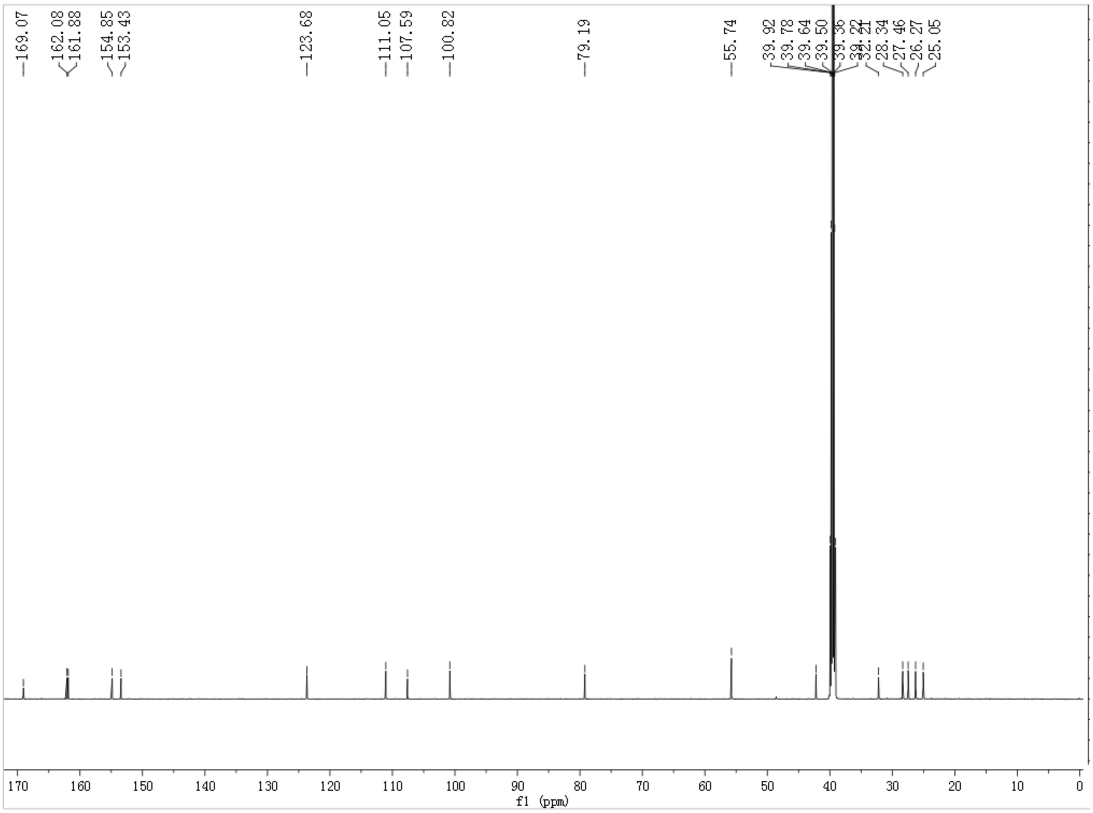

Fig. S14. The ^{13}C NMR spectrum for **10d**

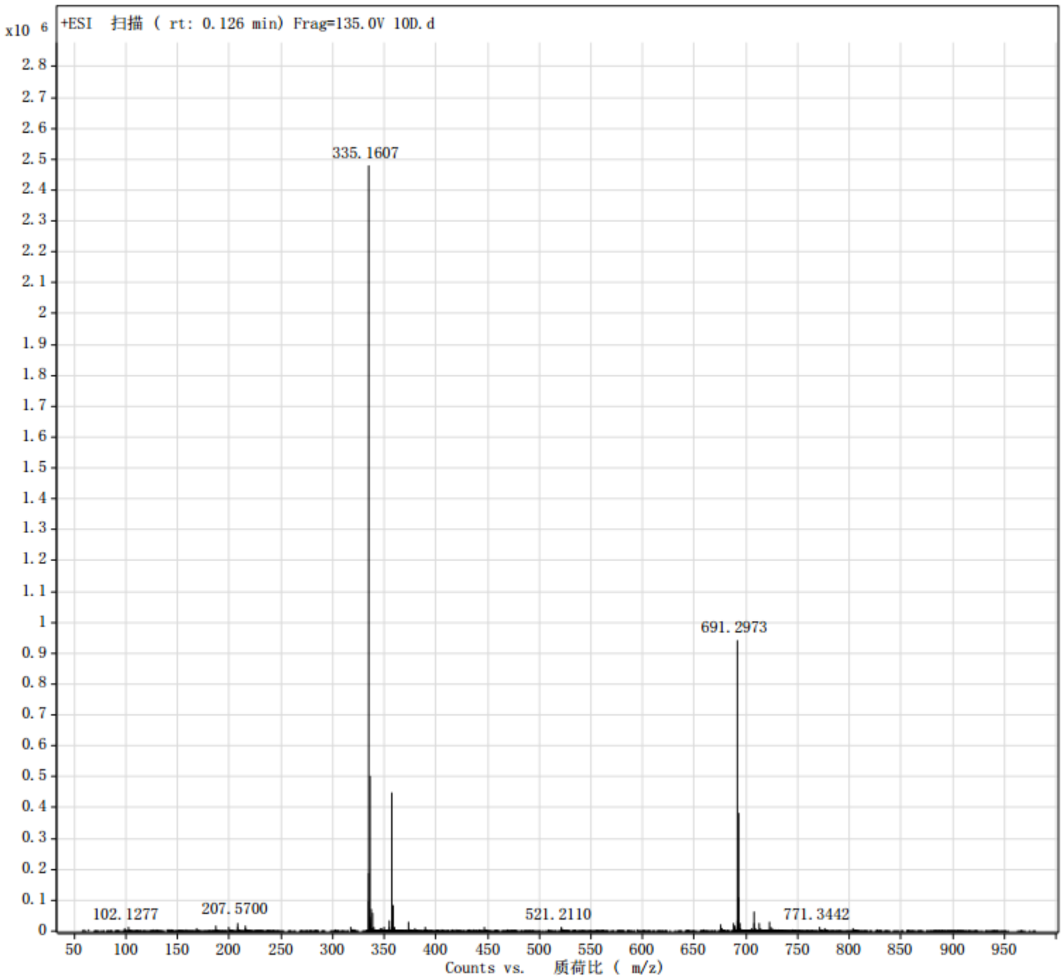

Fig. S15. The HR-ESIMS spectrum for **10d**

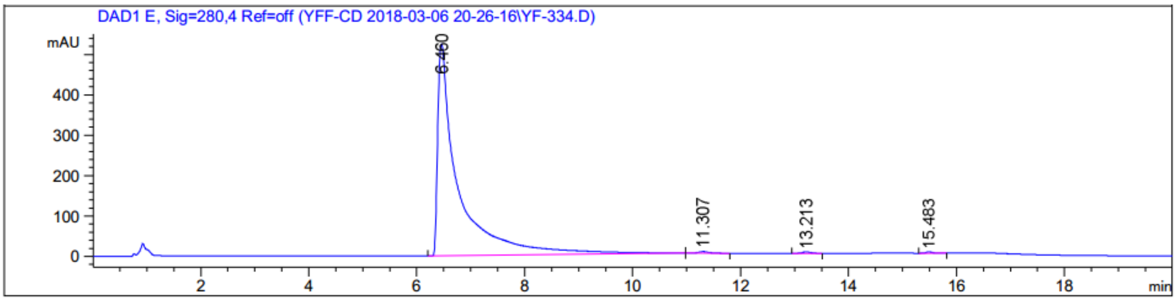

Peak #	RetTime [min]	Type	Width [min]	Area [mAU*s]	Height [mAU]	Area %
1	6.460	BB	0.3554	1.39743e4	523.53491	99.1252
2	11.307	BB	0.2131	56.87189	3.87854	0.4034
3	13.213	BB	0.1627	38.96036	3.53308	0.2764
4	15.483	BB	0.1217	27.49720	3.28385	0.1950

Fig. S16. The HPLC analysis for **10d**

Compound 10e

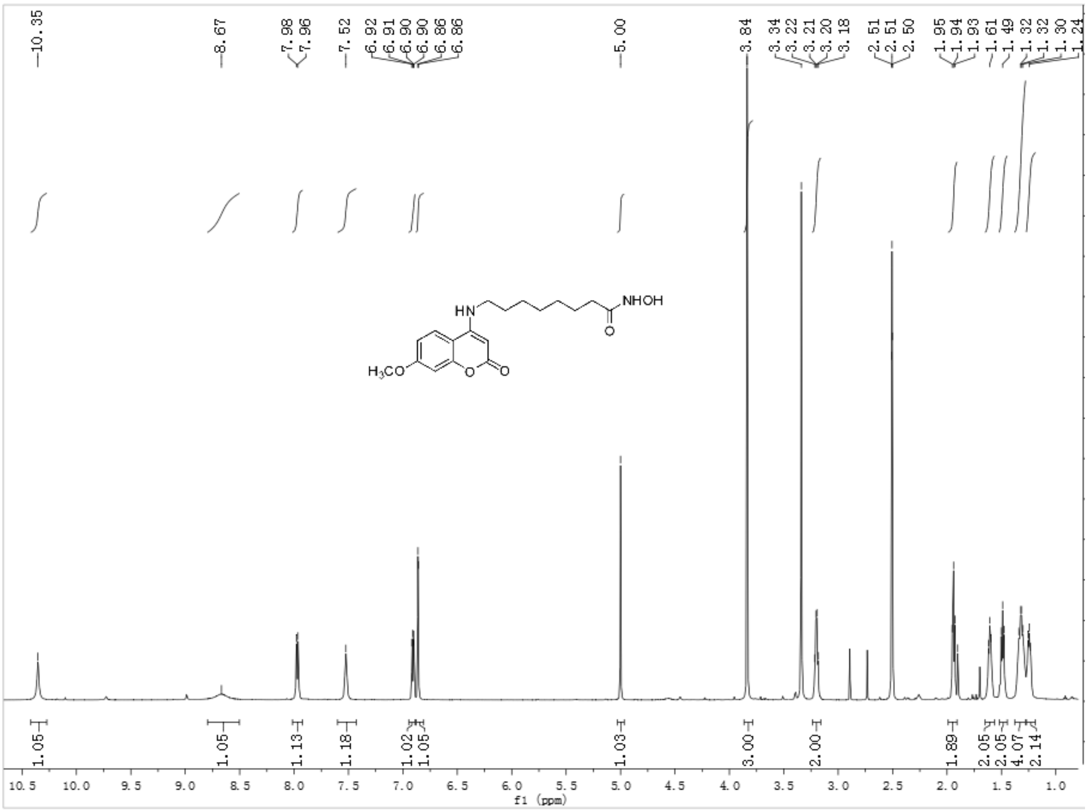

Fig. S17. The ^{1}H NMR spectrum for **10e**

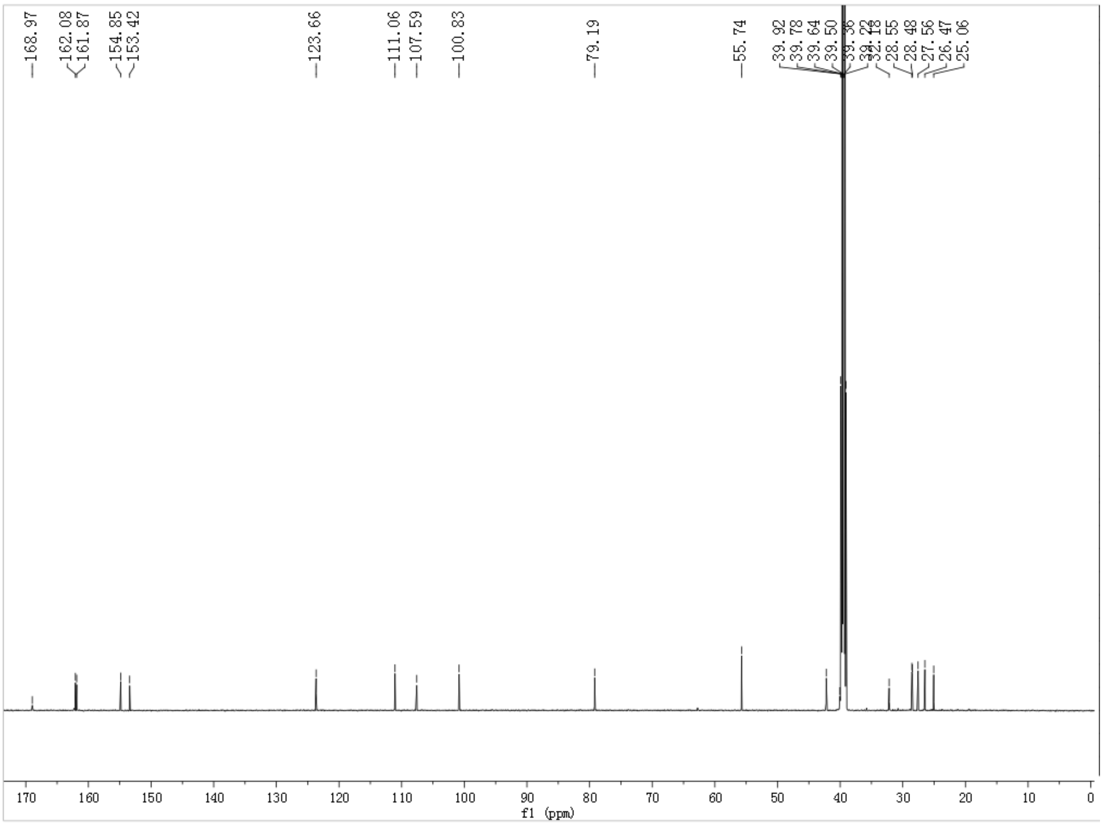

Fig. S18. The ^{13}C NMR spectrum for **10e**

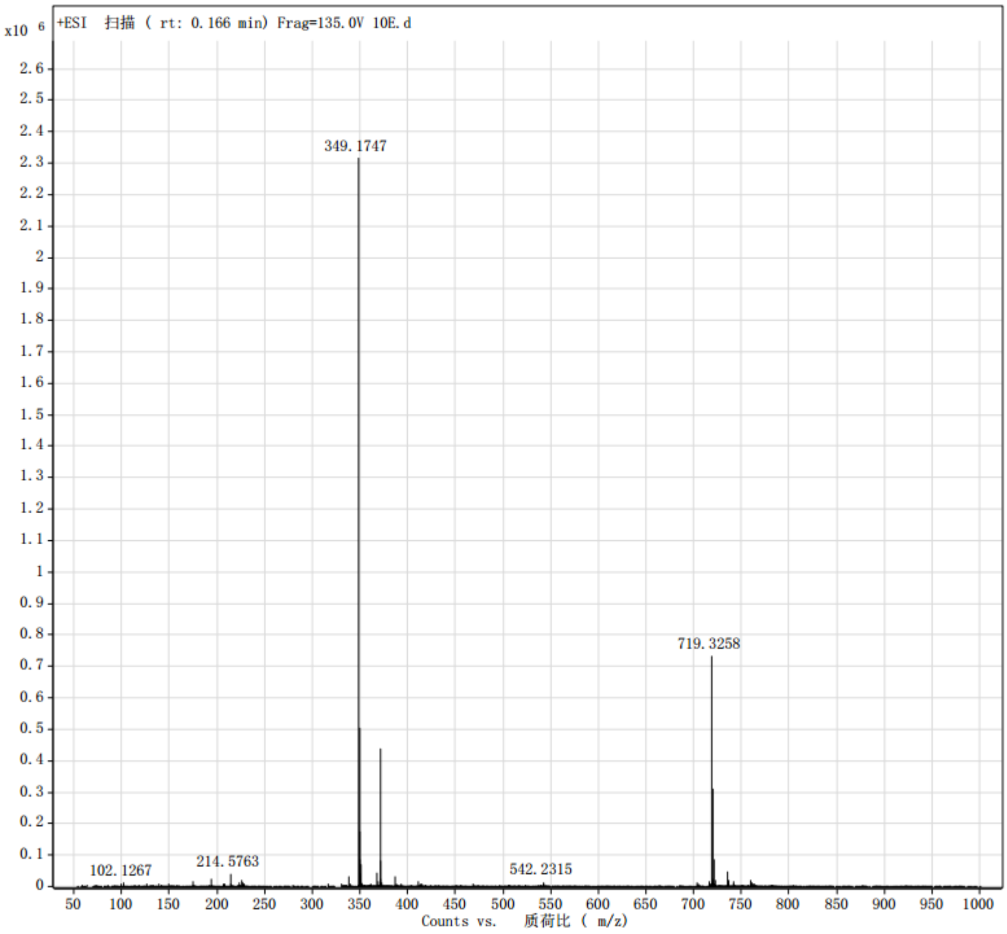

Fig. S19. The HR-ESIMS spectrum for **10e**

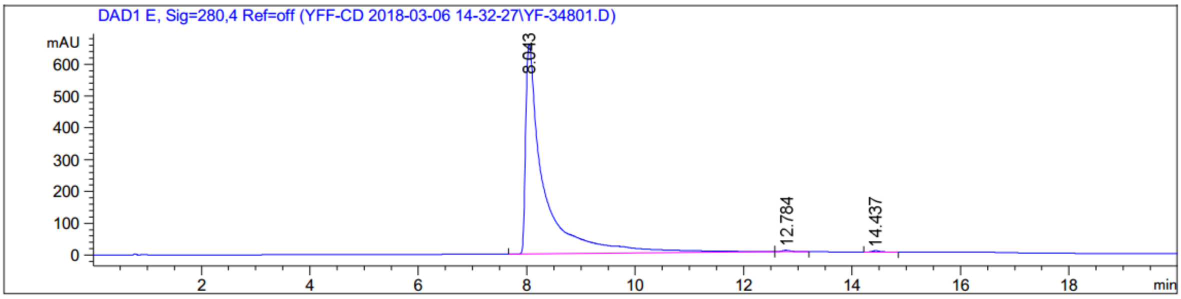

Peak #	RetTime [min]	Type	Width [min]	Area [mAU*s]	Height [mAU]	Area %
1	8.043	BB	0.3144	1.53982e4	659.52441	99.4262
2	12.784	BB	0.1648	47.42746	4.23173	0.3062
3	14.437	BB	0.1461	41.43515	4.23575	0.2675

Fig. S20. The HPLC analysis for **10e**

Compound 11a

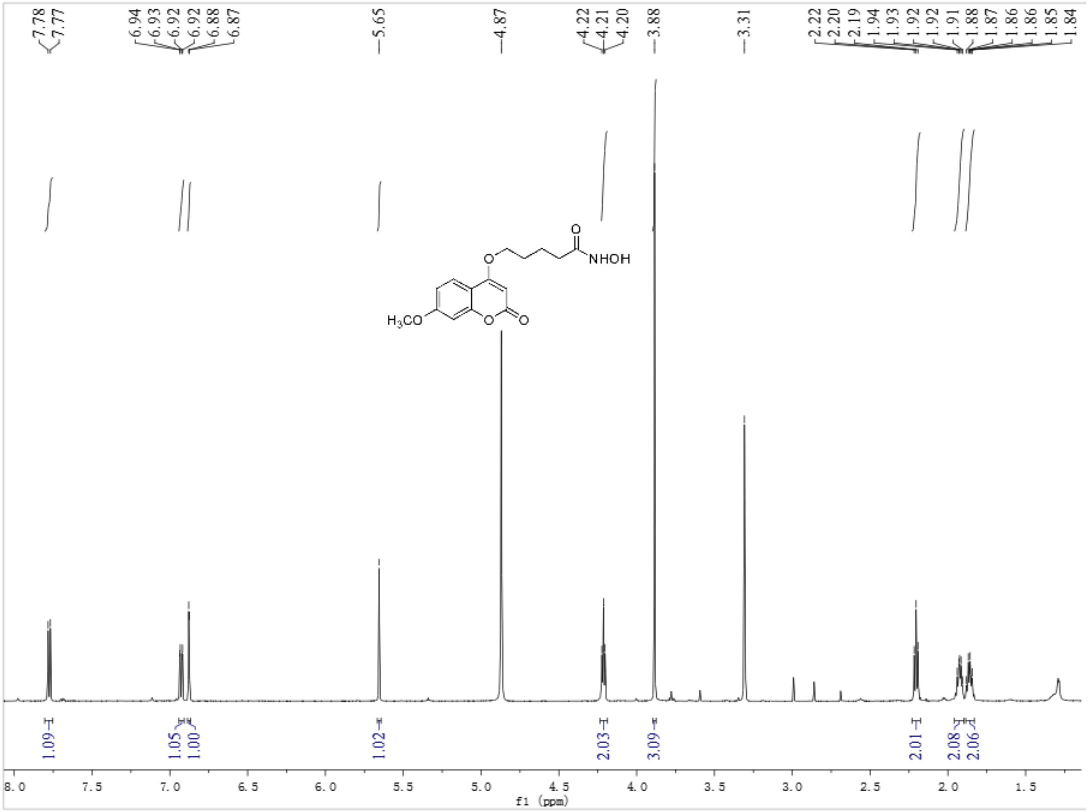

Fig. S21. The ^{1}H NMR spectrum for **11a**

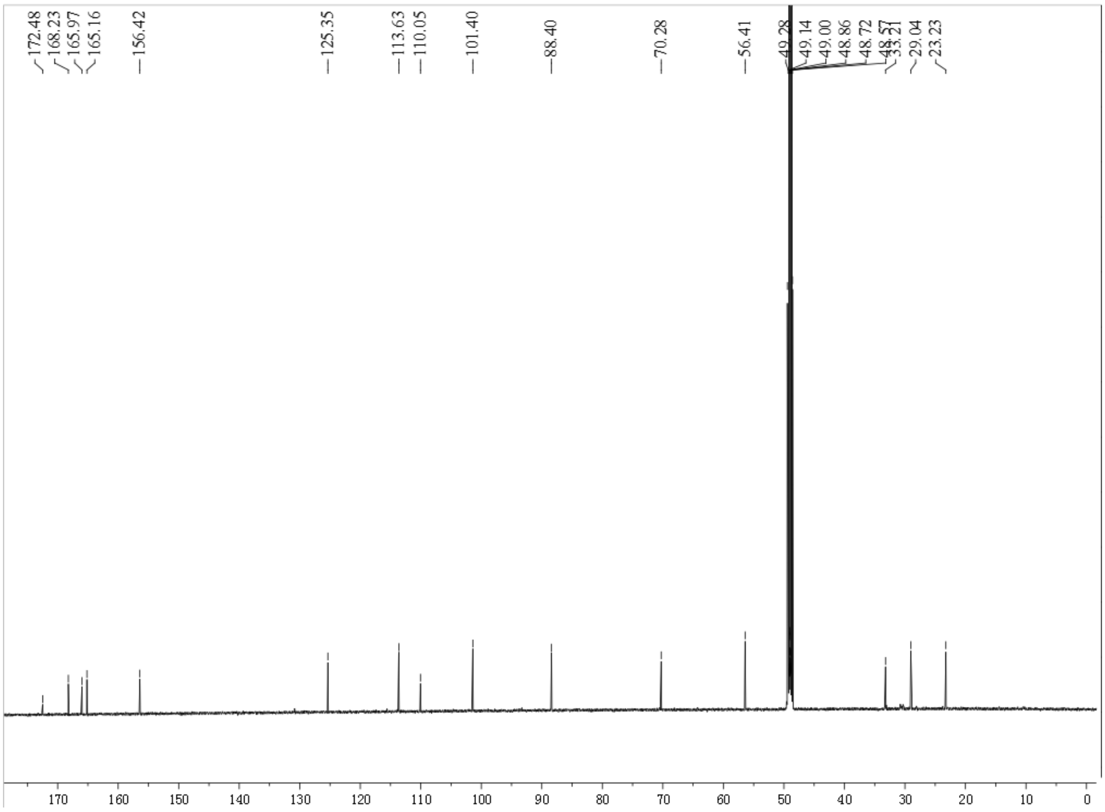

Fig. S22. The ^{13}C NMR spectrum for **11a**

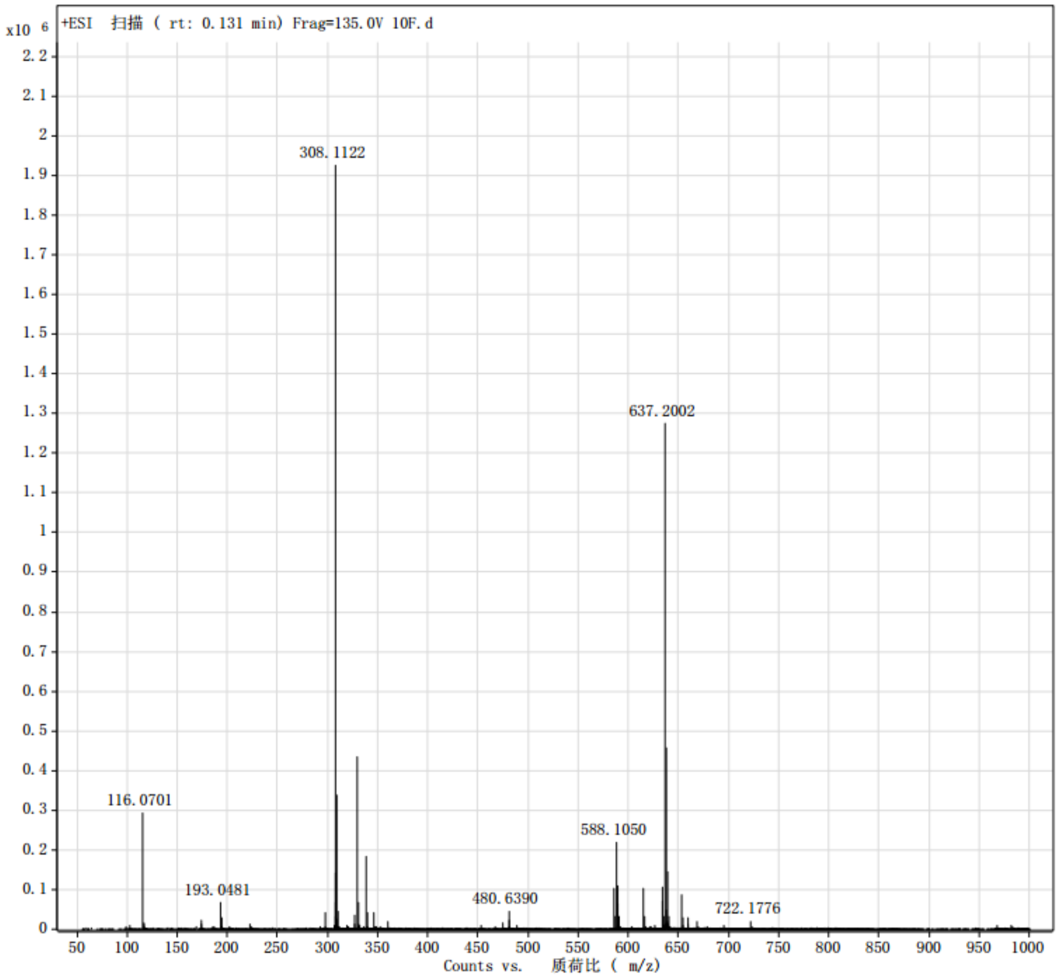

Fig. S23. The HR-ESIMS spectrum for **11a**

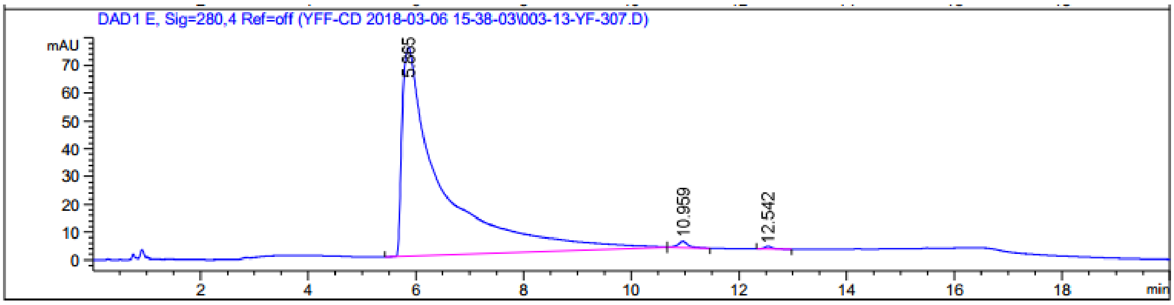

Peak #	RetTime [min]	Type	Width [min]	Area [mAU*s]	Height [mAU]	Area %
1	5.865	BB	0.6679	3807.21216	75.04779	98.9683
2	10.959	BB	0.1838	29.50382	2.29740	0.7670
3	12.542	BB	0.1470	10.18295	1.01550	0.2647

Fig. S24. The HPLC analysis for **11a**

Compound 11b

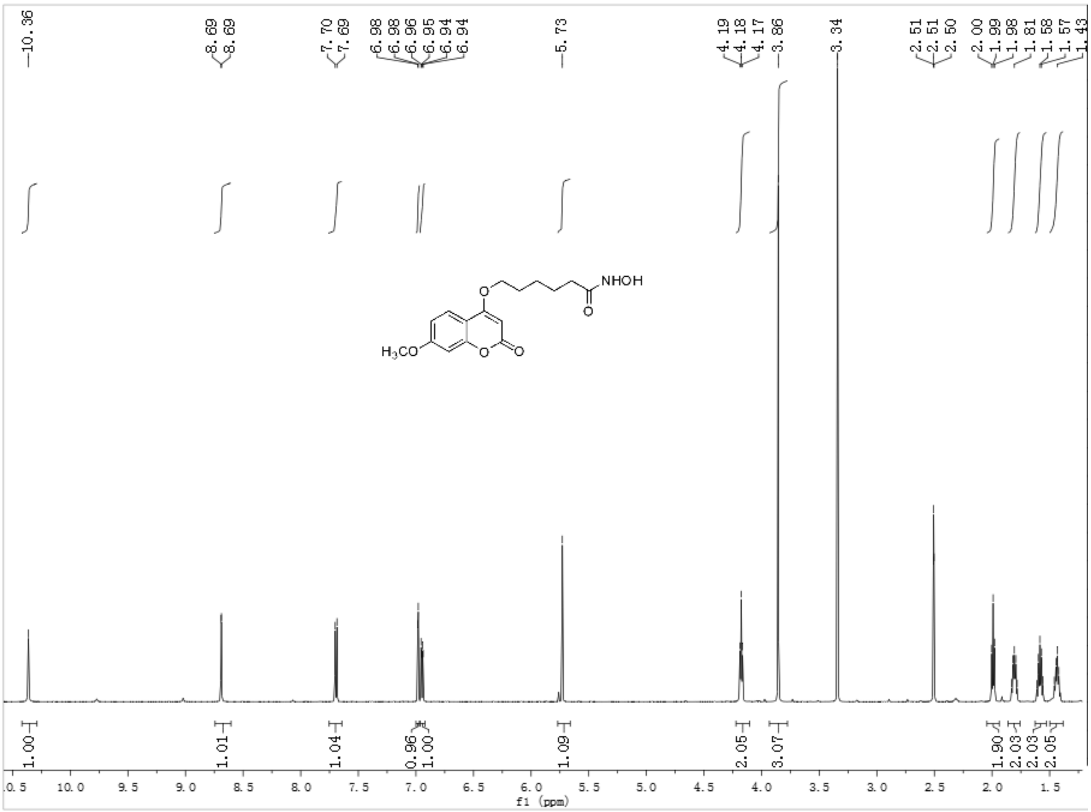

Fig. S25. The ^{1}H NMR spectrum for **11b**

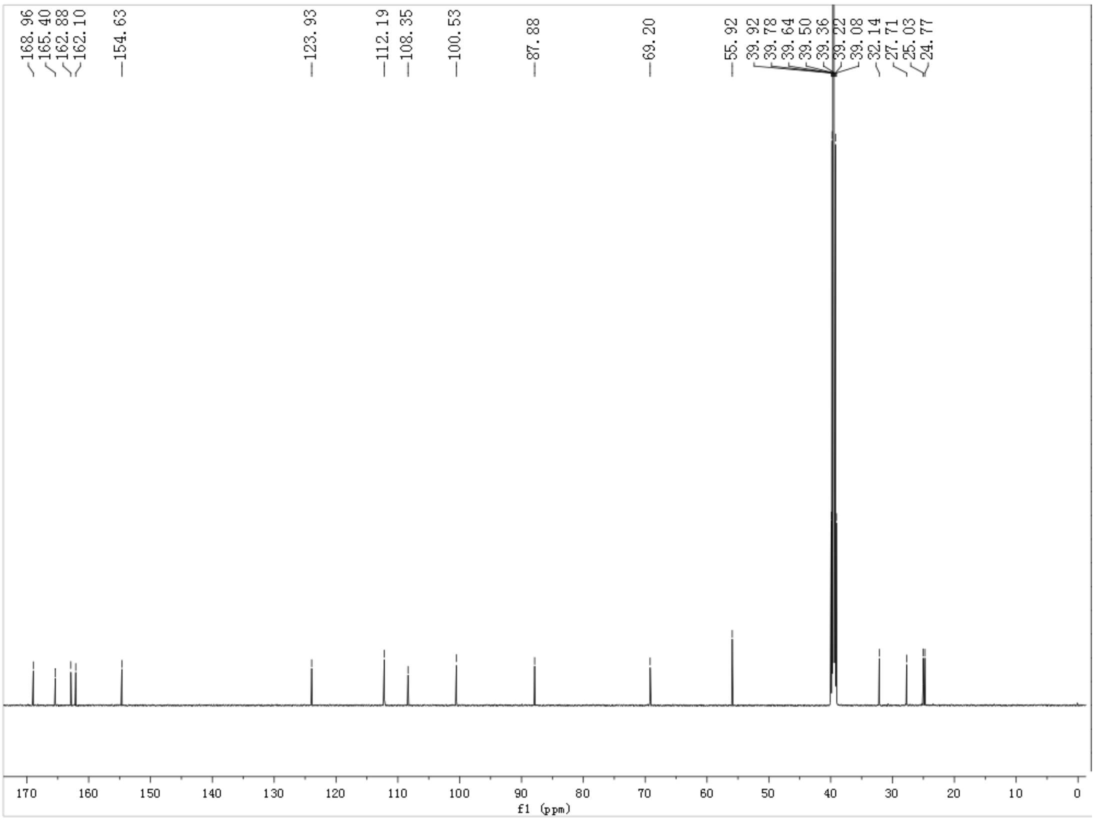

Fig. S26. The ^{13}C NMR spectrum for **11b**

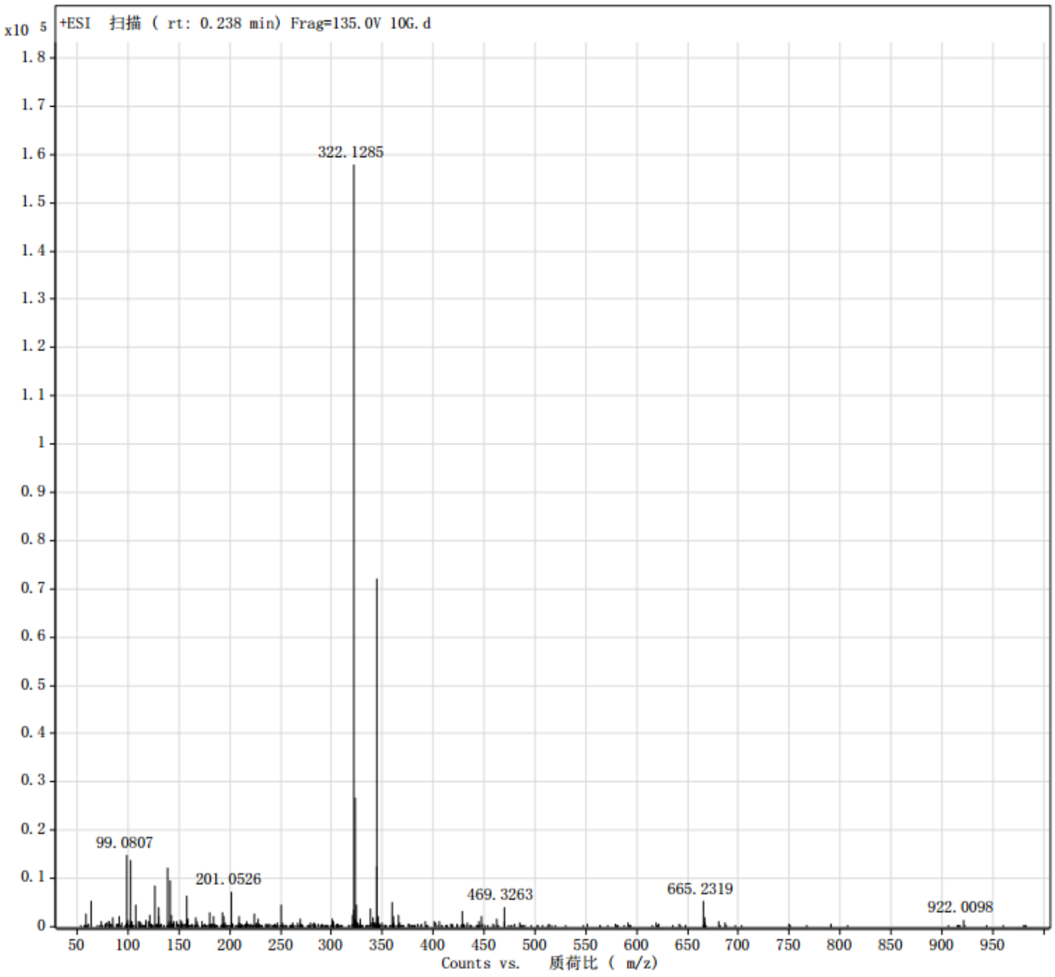

Fig. S27. The HR-ESIMS spectrum for **11b**

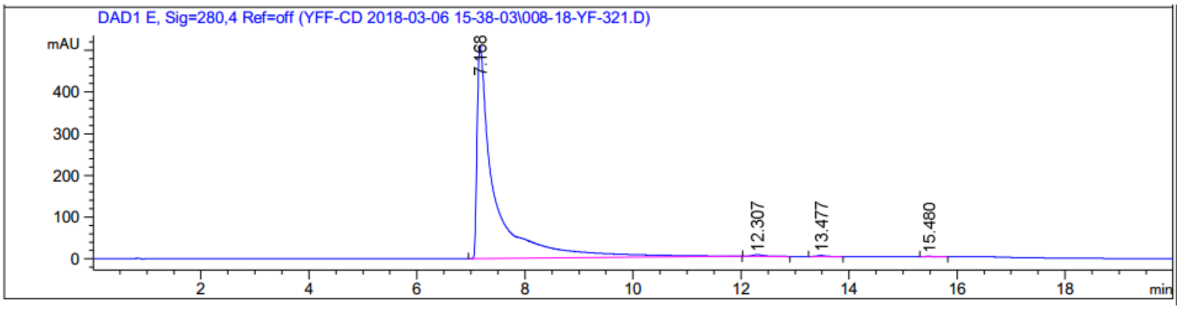

Peak #	RetTime [min]	Type	Width [min]	Area [mAU*s]	Height [mAU]	Area %
1	7.168	BB	0.3078	1.16255e4	510.30573	99.1898
2	12.307	BB	0.1959	52.95124	3.91351	0.4518
3	13.477	BB	0.1582	31.56357	2.91873	0.2693
4	15.480	BB	0.1627	10.44768	1.01020	0.0891

Fig. S28. The HPLC analysis for **11b**

Compound 11c

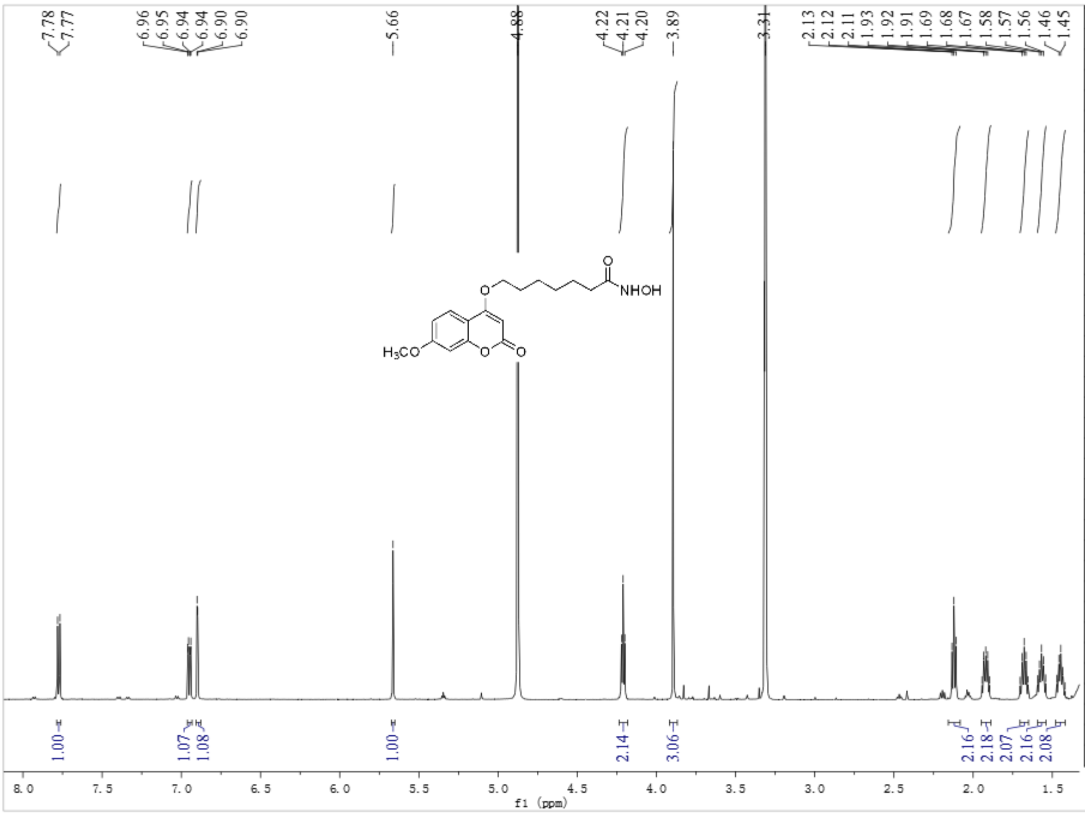

Fig. S29. The ^{1}H NMR spectrum for **11c**

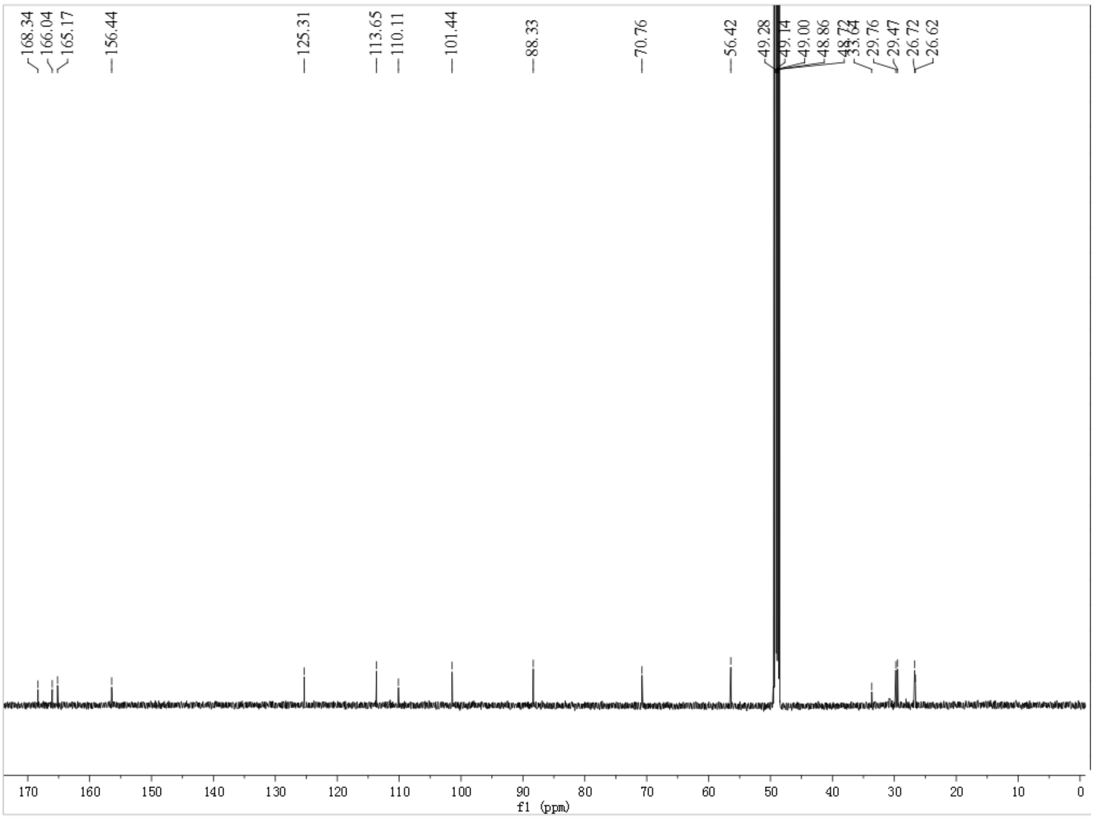

Fig. S30. The ^{13}C NMR spectrum for **11c**

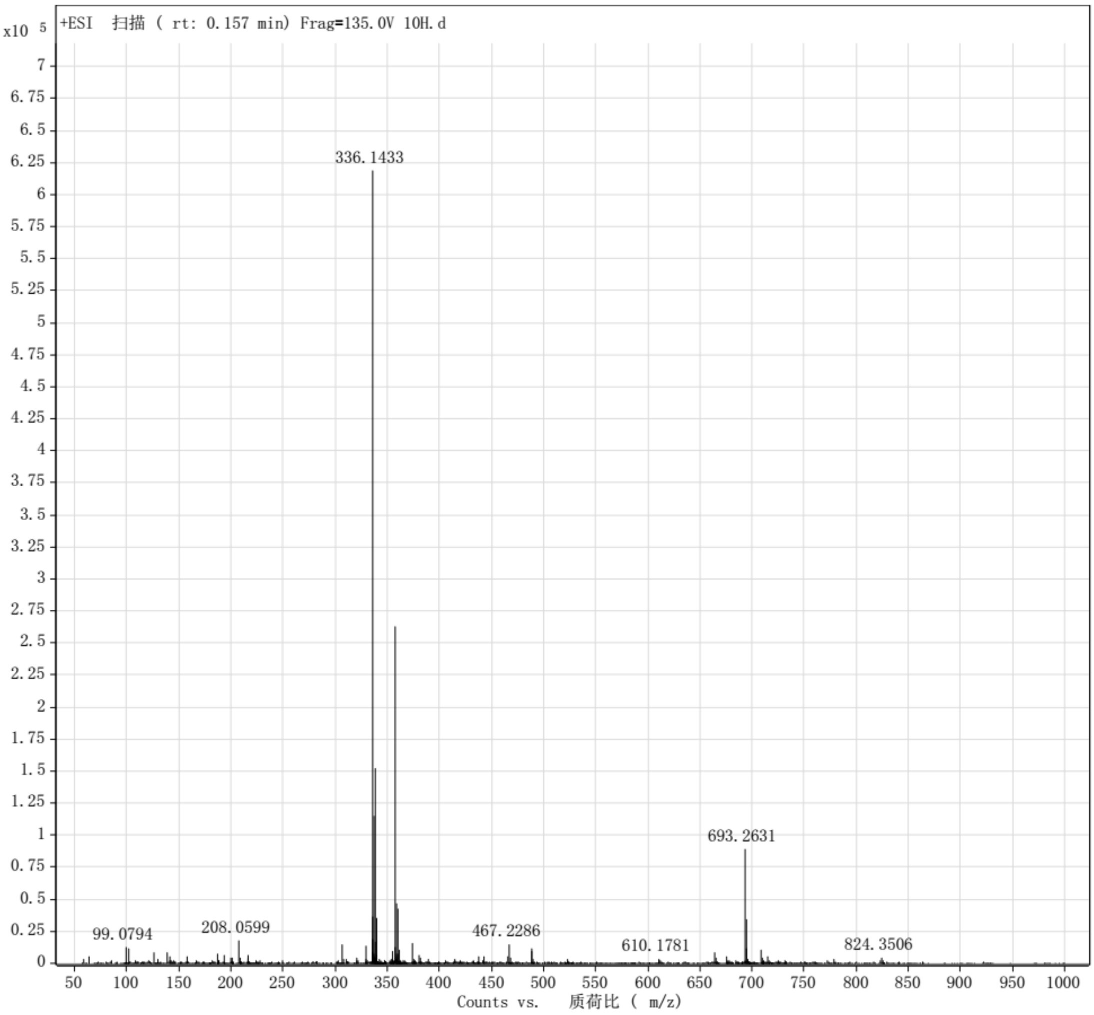

Fig. S31. The HR-ESIMS spectrum for **11c**

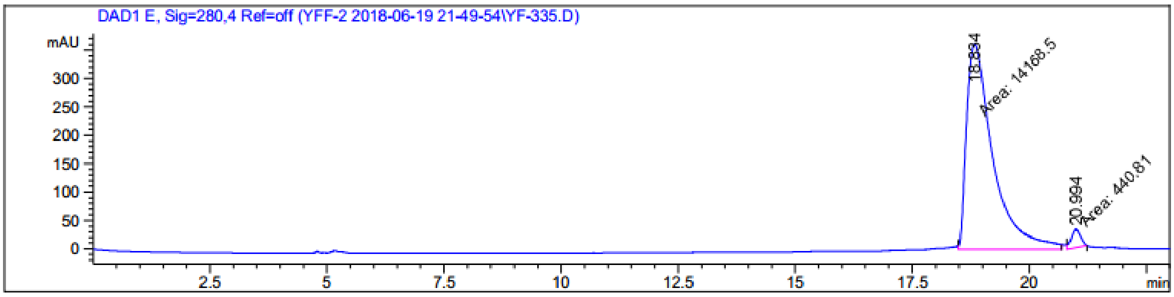

Peak #	RetTime [min]	Type	Width [min]	Area [mAU*s]	Height [mAU]	Area %
1	18.834	MM	0.6534	1.41685e4	361.37836	96.9827
2	20.994	MM	0.2297	440.81012	31.98563	3.0173

Fig. S32. The HPLC analysis for **11c**

Compound 11d

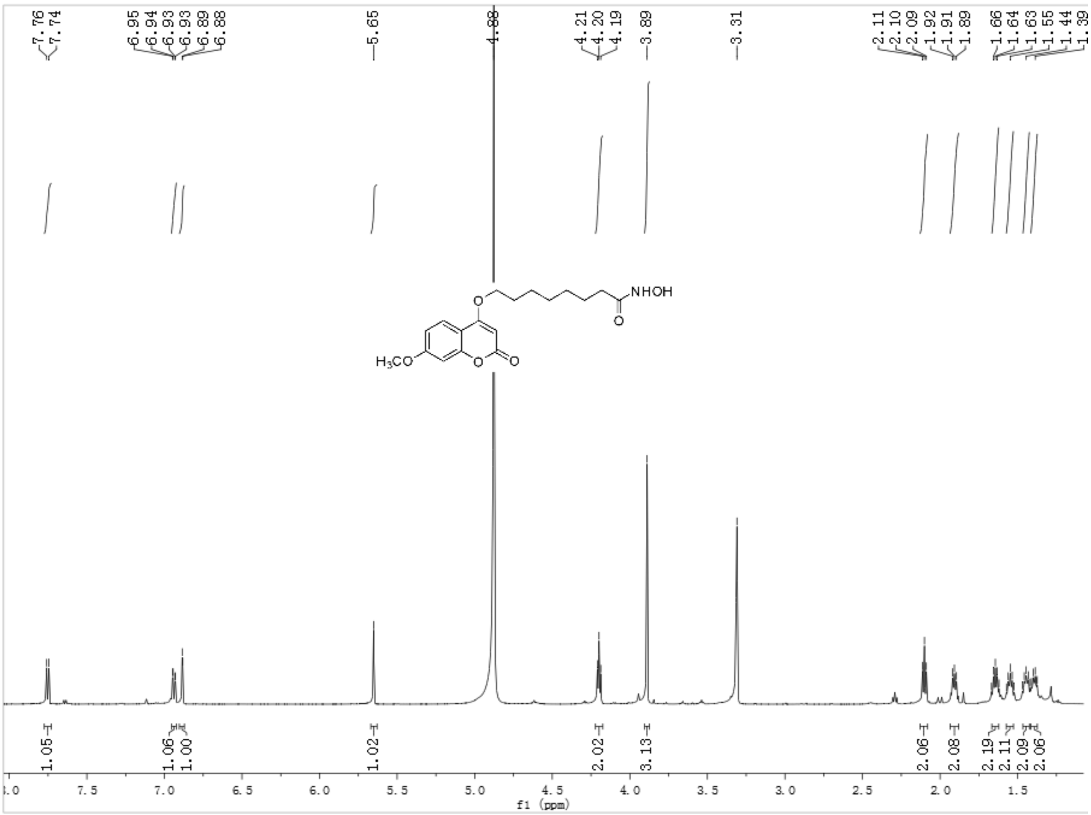

Fig. S33. The ^{1}H NMR spectrum for **11d**

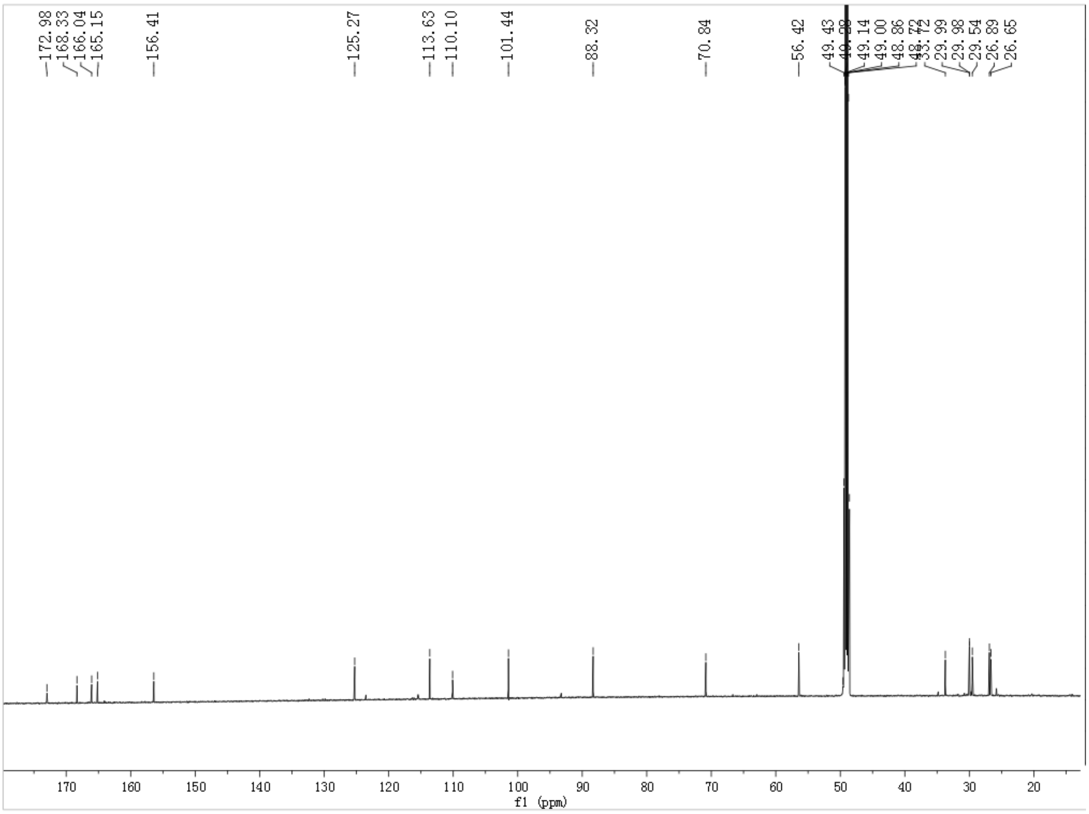

Fig. S34. The ^{13}C NMR spectrum for **11d**

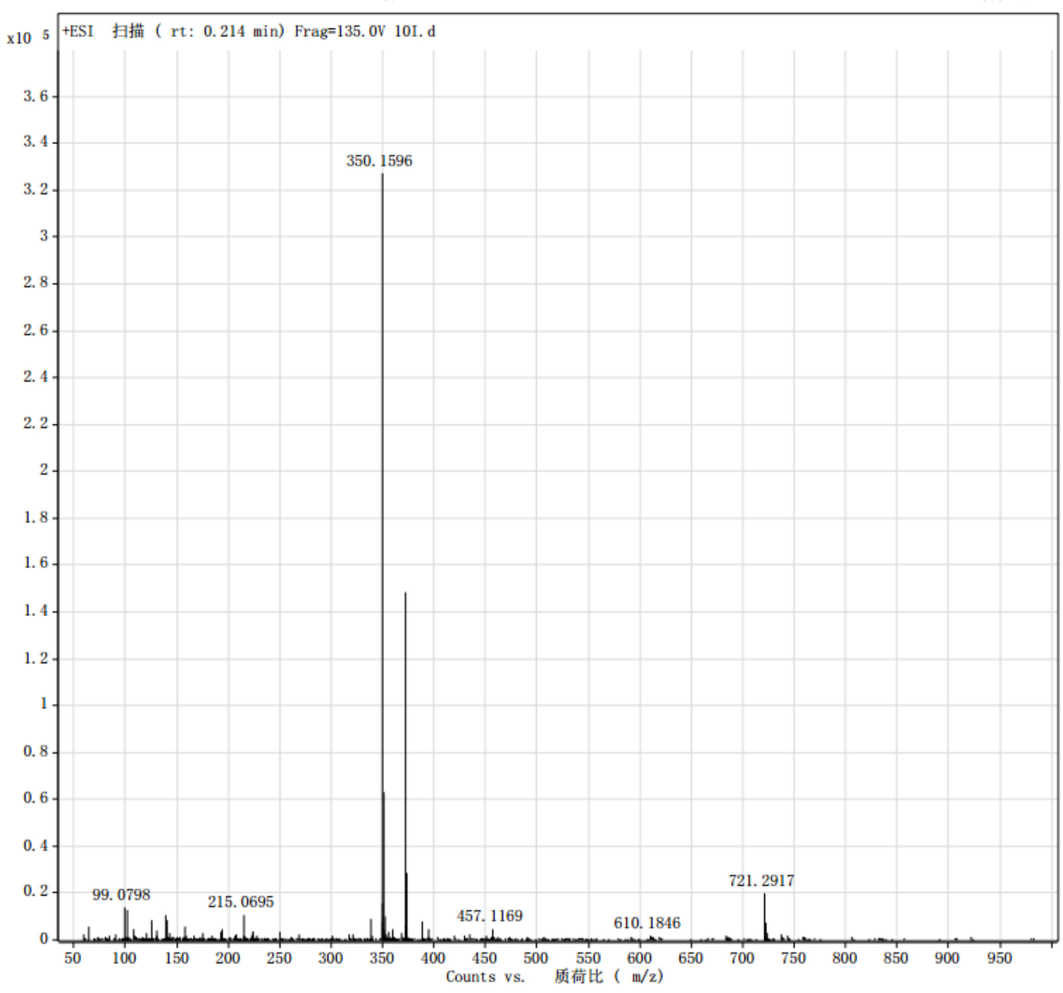

Fig. S35. The HR-ESIMS spectrum for **11d**

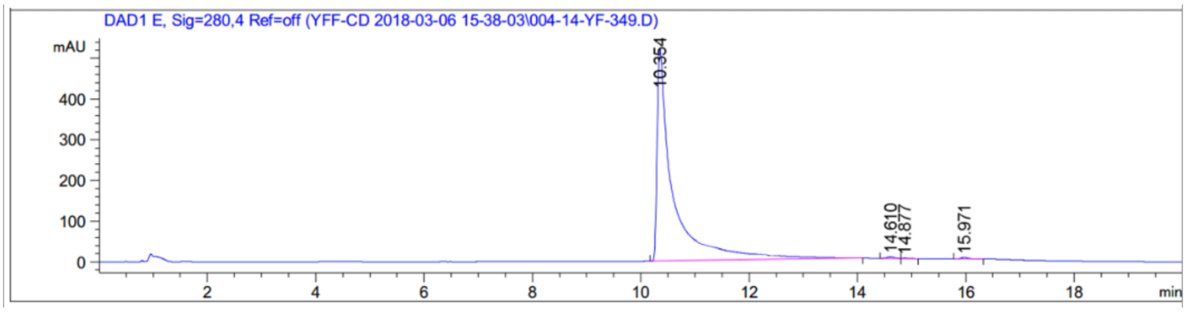

Peak #	RetTime [min]	Type	Width [min]	Area [mAU*s]	Height [mAU]	Area %
1	10.354	BV R	0.2746	1.09640e4	521.49292	99.2300
2	14.610	BV	0.1391	39.08996	4.25728	0.3538
3	14.877	VB	0.1085	8.27847	1.11673	0.0749
4	15.971	BB	0.1277	37.70428	4.41223	0.3412

Fig. S36. The HPLC analysis for **11d**

Compound 11e

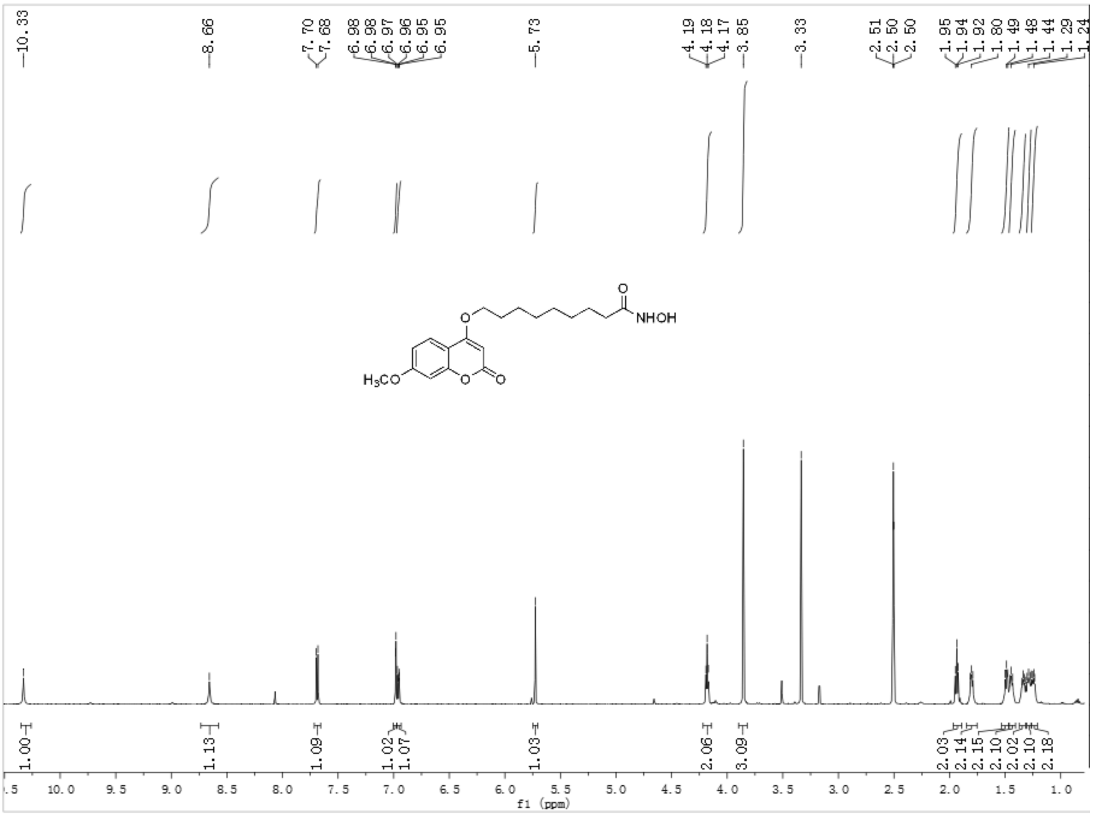

Fig. S37. The ^{1}H NMR spectrum for **11e**

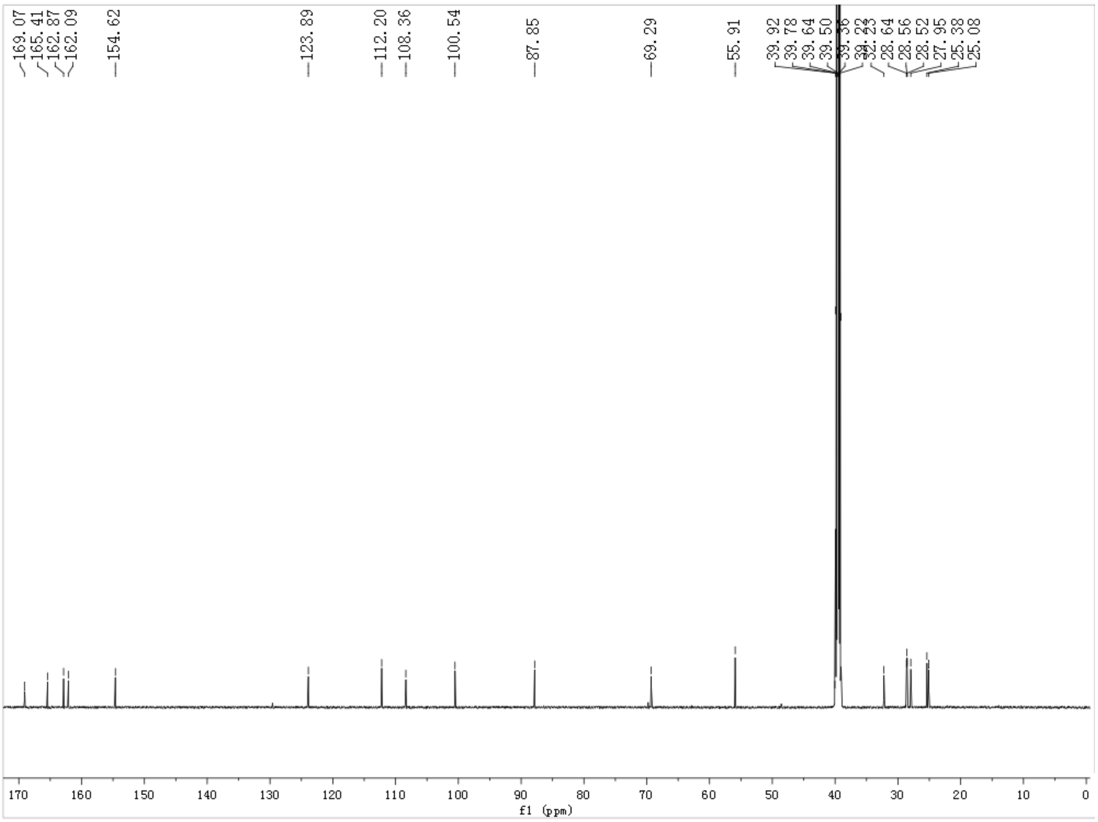

Fig. S38. The ^{13}C NMR spectrum for **11e**

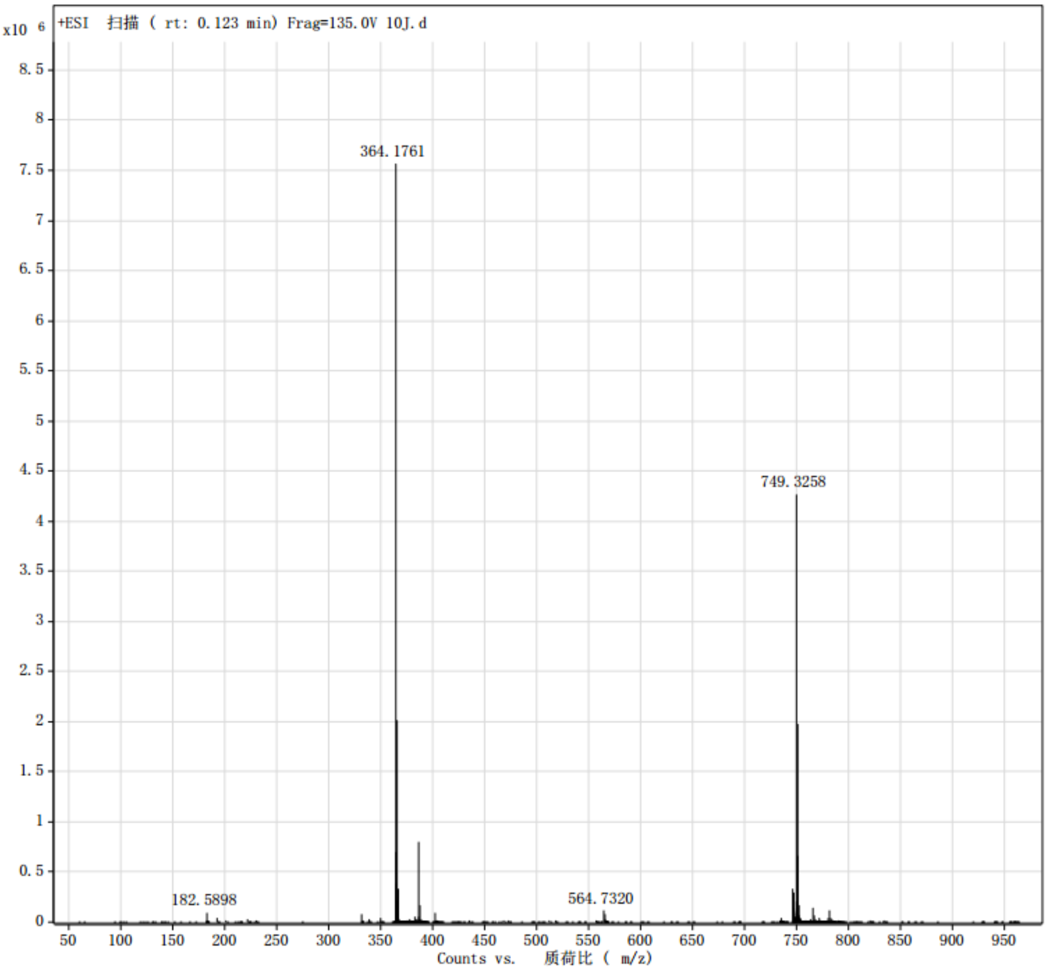

Fig. S39. The HR-ESIMS spectrum for **11e**

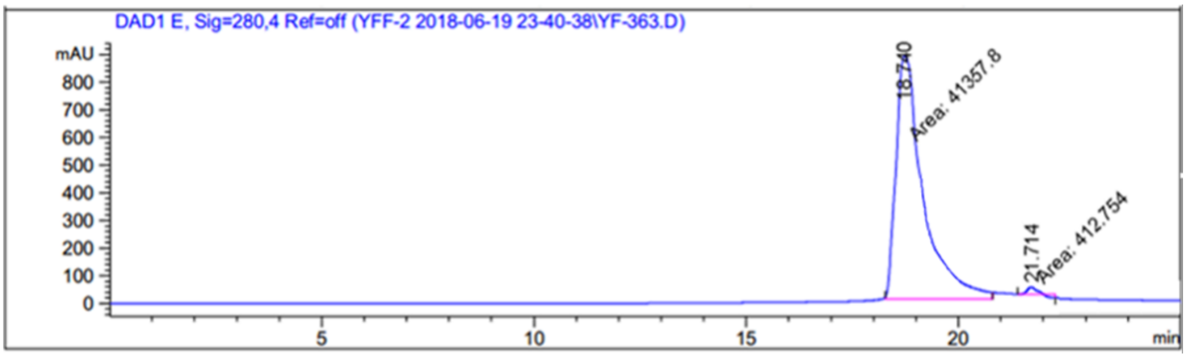

Peak #	RetTime [min]	Type	Width [min]	Area [mAU*s]	Height [mAU]	Area %
1	18.740	MM	0.7834	4.13578e4	879.92084	99.0119
2	21.714	MM	0.2614	412.75354	26.31210	0.9881

Fig. S40. The HPLC analysis for **11e**

Compound 12a

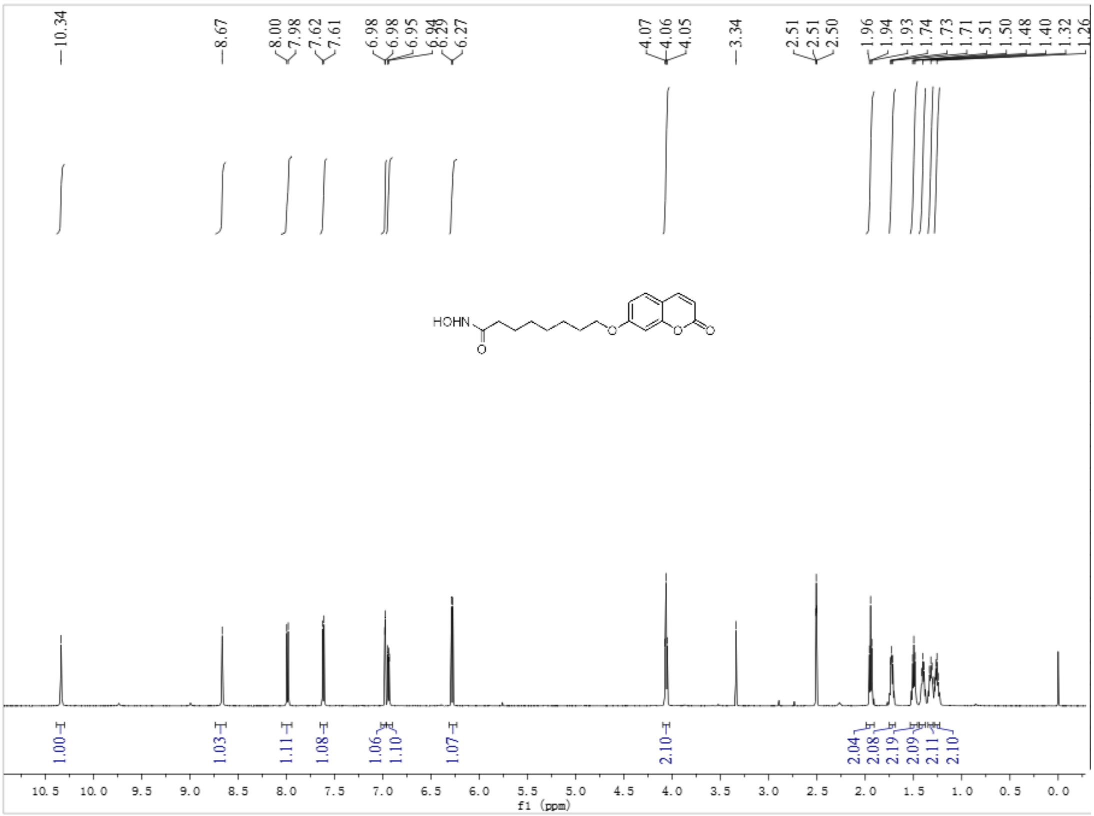

Fig. S41. The ^{1}H NMR spectrum for **12a**

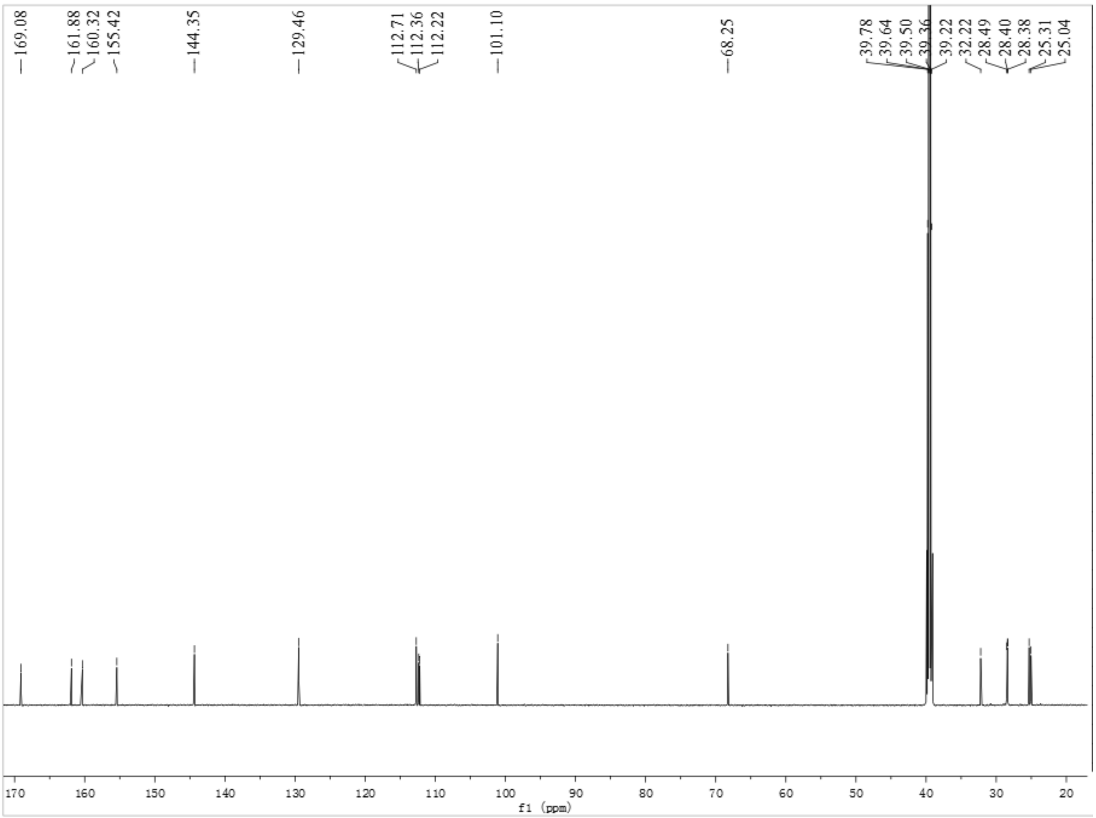

Fig. S42. The ^{13}C NMR spectrum for **12a**

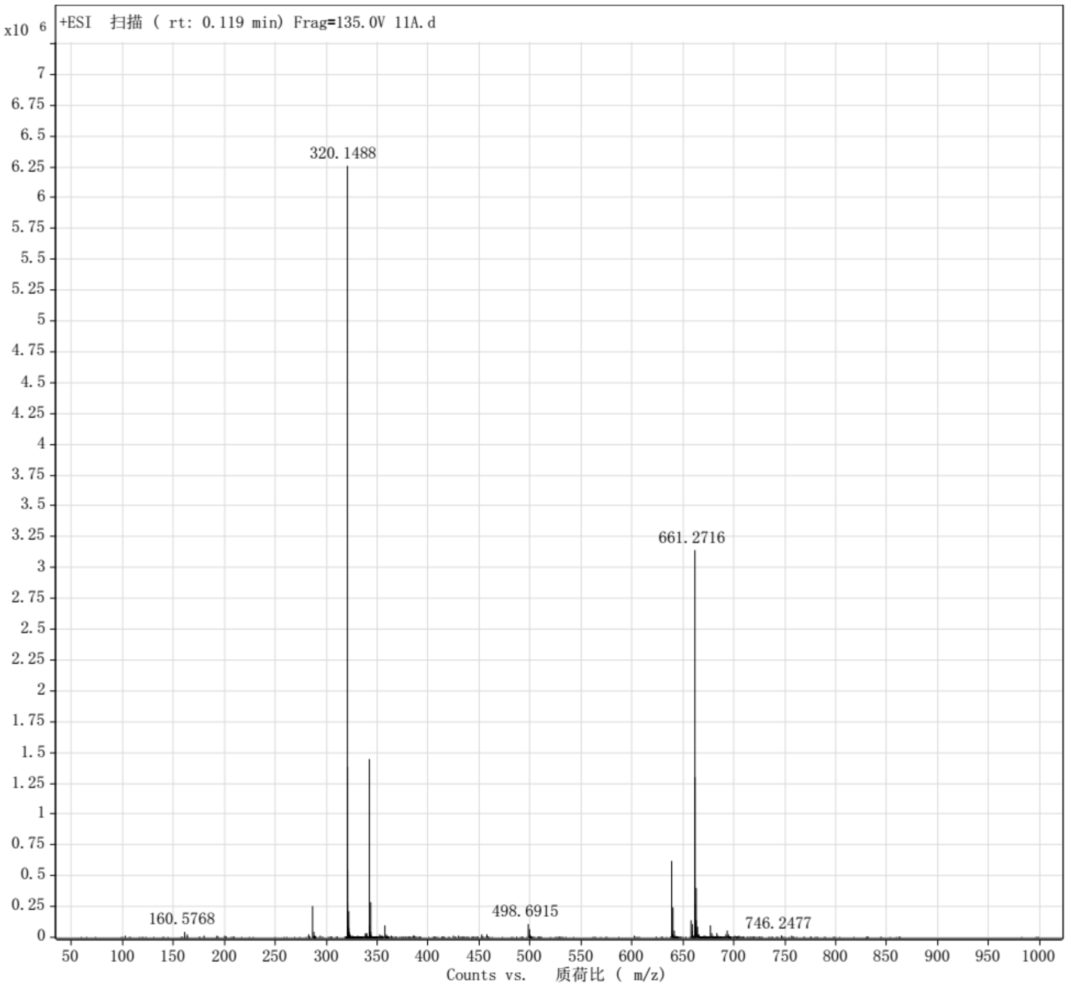

Fig. S43. The HR-ESIMS spectrum for**12a**

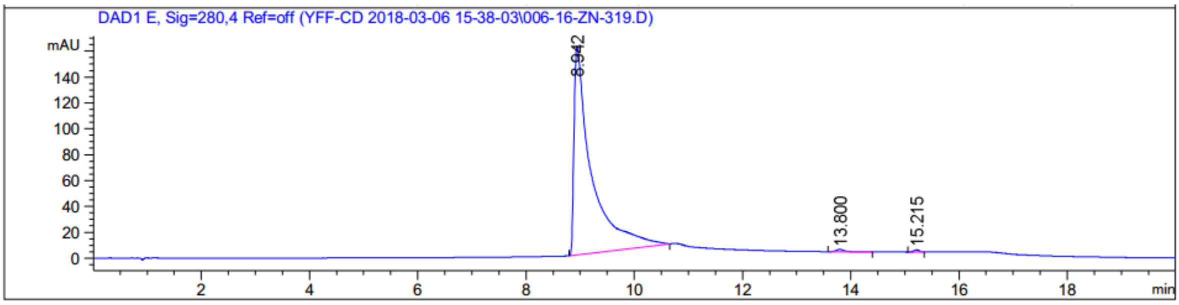

Peak #	RetTime [min]	Type	Width [min]	Area [mAU*s]	Height [mAU]	Area %
1	8.942	BB	0.3047	3625.35474	161.02577	99.1345
2	13.800	BB	0.1618	20.48607	1.84086	0.5602
3	15.215	BB	0.1130	11.16416	1.53199	0.3053

Fig. S44. The HPLC analysis for **12a**

Compound 12b

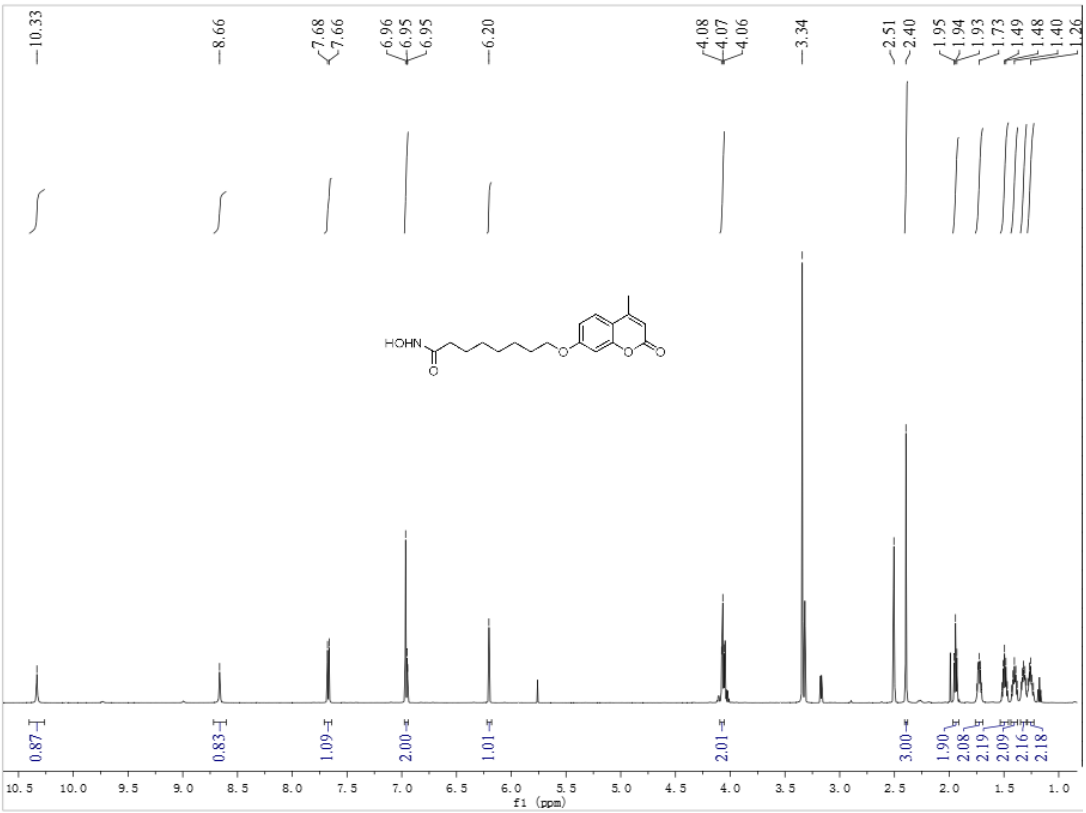

Fig. S45. The ^{1}H NMR spectrum for **12b**

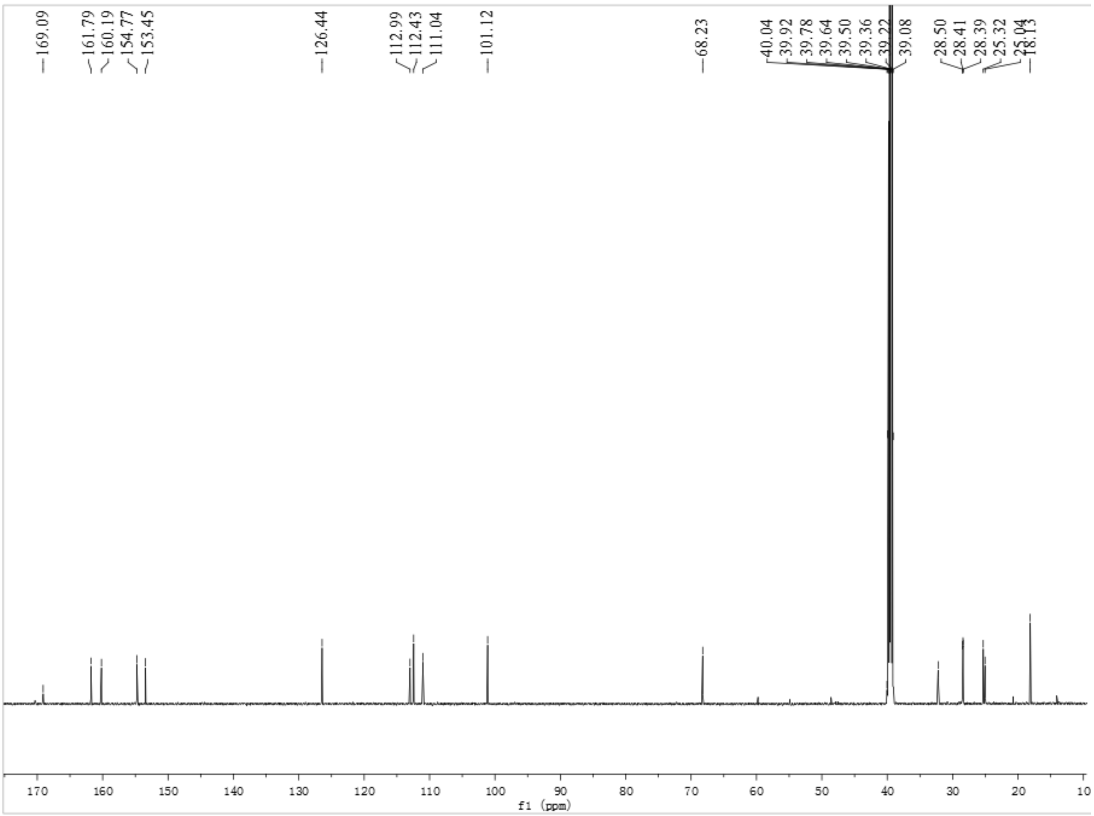

Fig. S46. The ^{13}C NMR spectrum for **12b**

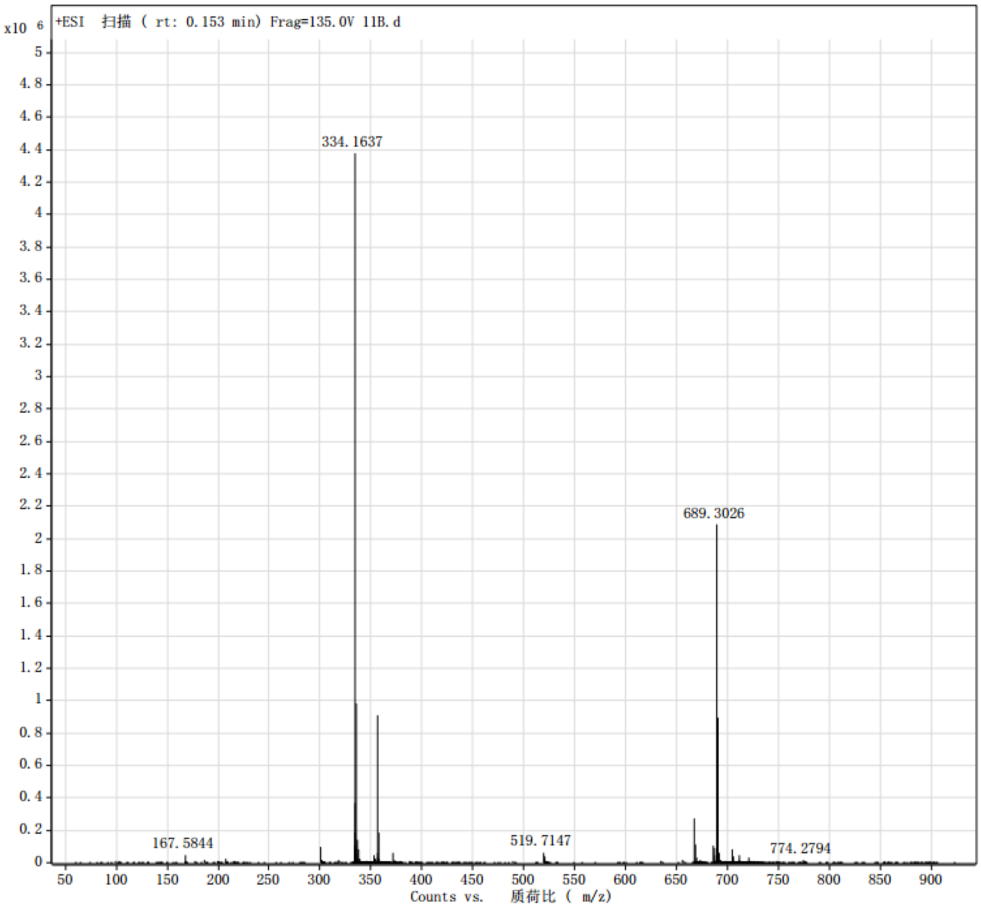

Fig. S47. The HR-ESIMS spectrum for **12b**

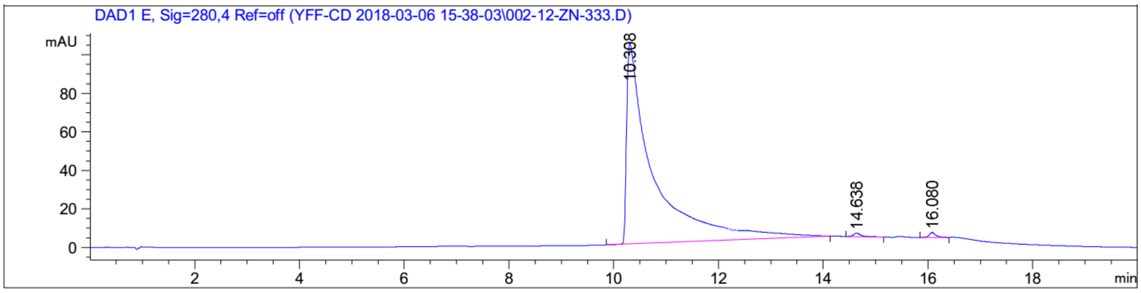

Peak #	RetTime [min]	Type	Width [min]	Area [mAU*s]	Height [mAU]	Area %
1	10.308	BV R	0.4694	3729.55029	104.24575	98.8427
2	14.638	BB	0.1648	21.15488	1.83029	0.5607
3	16.080	BB	0.1274	22.51370	2.58970	0.5967

Fig. S48. The HPLC analysis for **12b**

HDAC inhibition activity of compounds 10b-e, 11a-e and 12a-b

10b IC_{50} on HDAC1

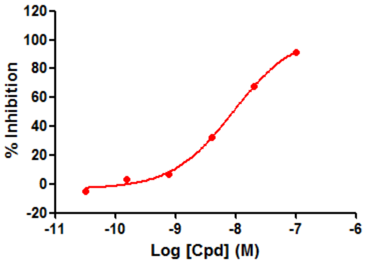

10c IC_{50} on HDAC1

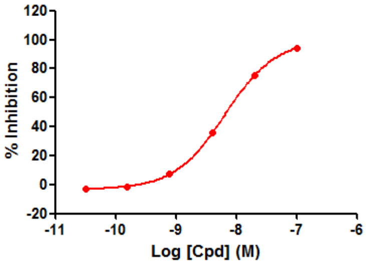

10d IC_{50} on HDAC1

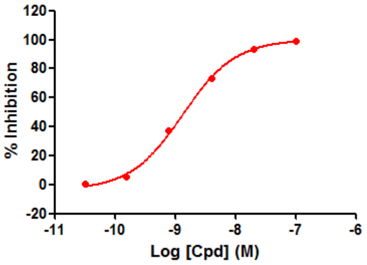

10e IC_{50} on HDAC1

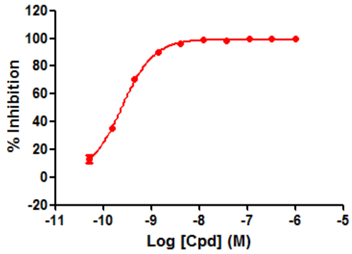

11a IC_{50} on HDAC1

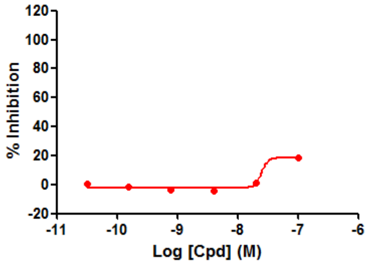

11b IC_{50} on HDAC1

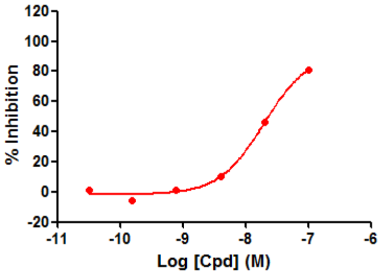

11c IC_{50} on HDAC1

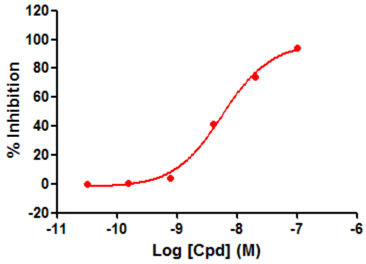

11d IC_{50} on HDAC1

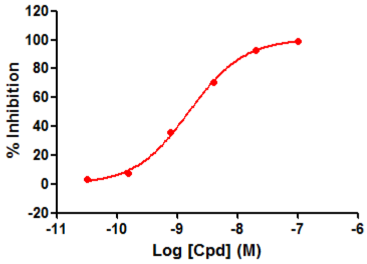

11e IC_{50} on HDAC1

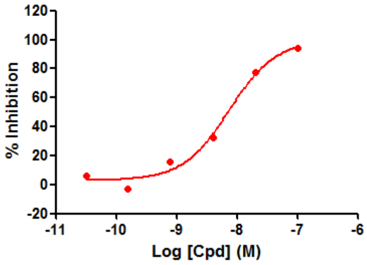

12a IC_{50} on HDAC1

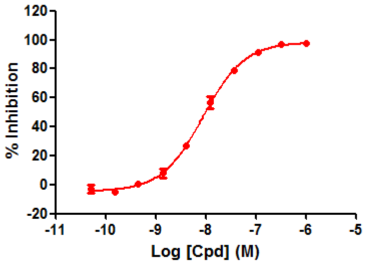

12b IC_{50} on HDAC1

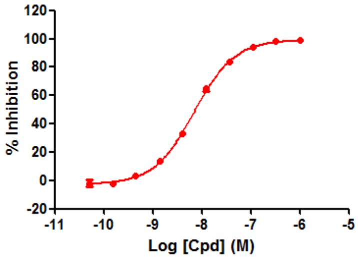

SAHA IC_{50} on HDAC1

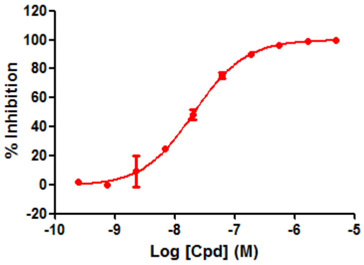

Inhibition activity (IC_{50}) of compounds 10e and 11d on different HDAC isoforms

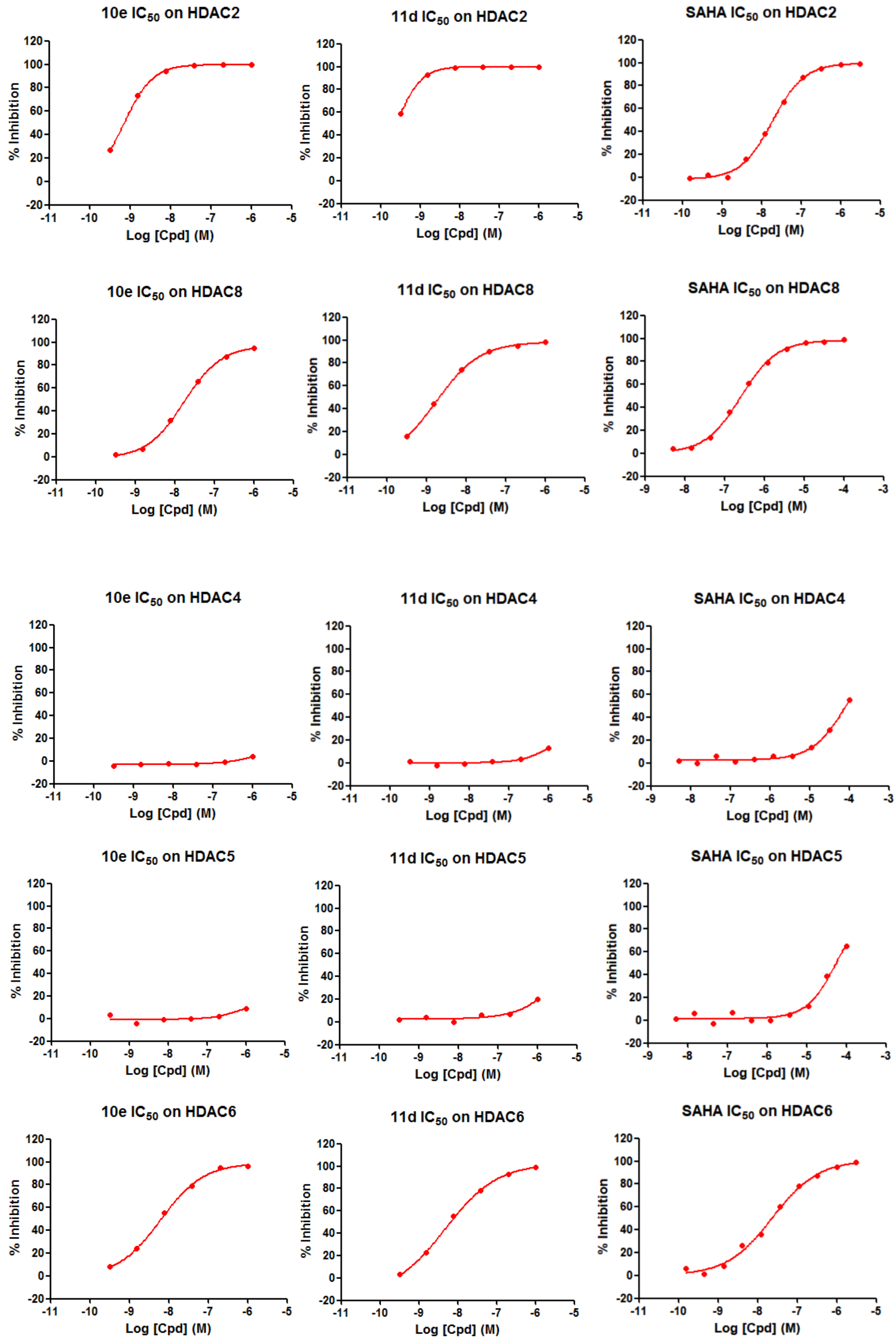

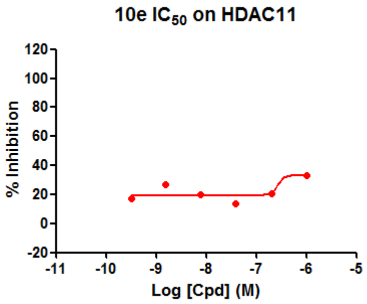
10e IC50 on HDAC11
% Inhibition
Log [Cpd] (M)

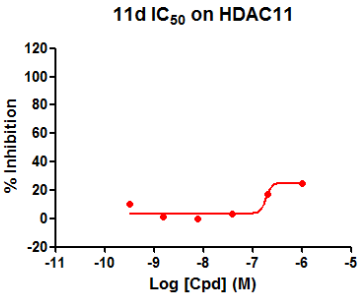
11d IC50 on HDAC11
% Inhibition
Log [Cpd] (M)

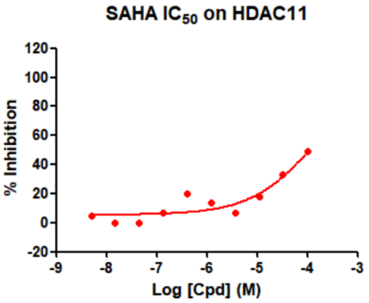
SAHA IC50 on HDAC11
% Inhibition
Log [Cpd] (M)

Anti-proliferative activities of compounds 10e and 11d against different cancer cell lines

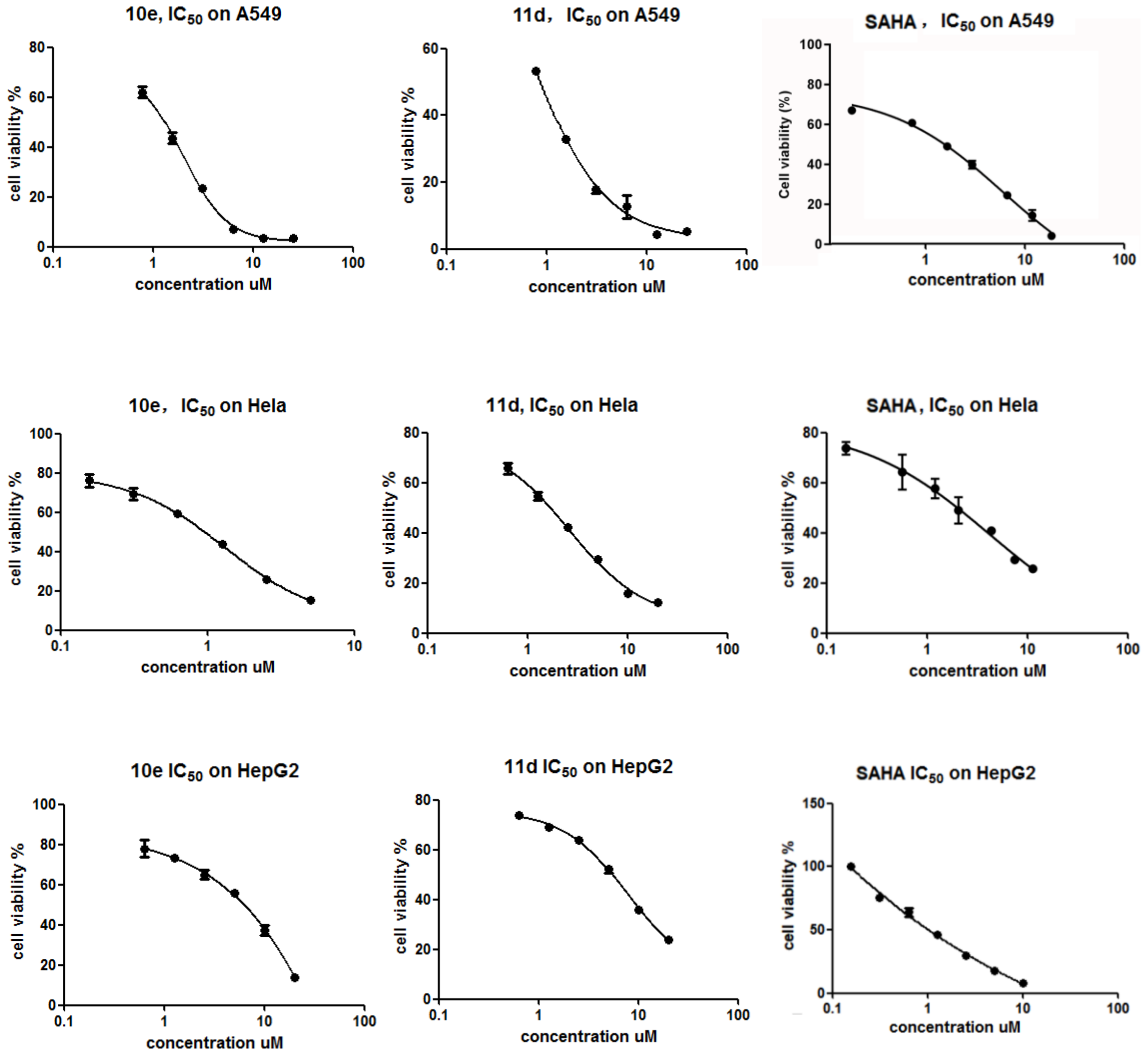

Supplement: Supplementary file 1 [file molecules-24-02569-s001.pdf]
